# Effect of a four-week virtual reality-based training versus conventional therapy on upper limb motor function after stroke: A multicenter parallel group randomized trial

**DOI:** 10.1371/journal.pone.0204455

**Published:** 2018-10-24

**Authors:** Corina Schuster-Amft, Kynan Eng, Zorica Suica, Irene Thaler, Sandra Signer, Isabelle Lehmann, Ludwig Schmid, Michael A. McCaskey, Miura Hawkins, Martin L. Verra, Daniel Kiper

**Affiliations:** 1 Research Department, Reha Rheinfelden, Rheinfelden, Switzerland; 2 Institute for Rehabilitation and Performance Technology, Bern University of Applied Sciences, Burgdorf, Switzerland; 3 Institute of Neuroinformatics, University of Zurich and ETH Zurich, Zurich, Switzerland; 4 Department of Physiotherapy, Insel Group, Bern University Hospital, Berne, Switzerland; 5 Physiotherapy Department, Buergerspital Solothurn, Solothurn, Switzerland; 6 Physiotherapy Department, Zurcher RehaZentrum Lengg, Zurich, Switzerland; 7 Institute of Human Movement Sciences, ETH Zurich, Zurich, Switzerland; TNO, NETHERLANDS

## Abstract

**Background:**

Virtual reality-based training has found increasing use in neurorehabilitation to improve upper limb training and facilitate motor recovery.

**Objective:**

The aim of this study was to directly compare virtual reality-based training with conventional therapy.

**Methods:**

In a multi-center, parallel-group randomized controlled trial, patients at least 6 months after stroke onset were allocated either to an experimental group (virtual reality-based training) or a control group receiving conventional therapy (16x45 minutes within 4 weeks). The virtual reality-based training system replicated patients´ upper limb movements in real-time to manipulate virtual objects.

Blinded assessors tested patients twice before, once during, and twice after the intervention up to 2-month follow-up for dexterity (primary outcome: Box and Block Test), bimanual upper limb function (Chedoke-McMaster Arm and Hand Activity Inventory), and subjective perceived changes (Stroke Impact Scale).

**Results:**

54 eligible patients (70 screened) participated (15 females, mean age 61.3 years, range 20–81 years, time since stroke 3.0±SD 3 years). 22 patients were allocated to the experimental group and 32 to the control group (3 drop-outs). Patients in the experimental and control group improved: Box and Block Test mean 21.5±SD 16 baseline to mean 24.1±SD 17 follow-up; Chedoke-McMaster Arm and Hand Activity Inventory mean 66.0±SD 21 baseline to mean 70.2±SD 19 follow-up. An intention-to-treat analysis found no between-group differences.

**Conclusions:**

Patients in the experimental and control group showed similar effects, with most improvements occurring in the first two weeks and persisting until the end of the two-month follow-up period. The study population had moderate to severely impaired motor function at entry (Box and Block Test mean 21.5±SD 16). Patients, who were less impaired (Box and Block Test range 18 to 72) showed higher improvements in favor of the experimental group. This result could suggest that virtual reality-based training might be more applicable for such patients than for more severely impaired patients.

**Trial registration:**

ClinicalTrials.gov NCT01774669.

## Introduction

Virtual reality-based rehabilitation systems are gaining popularity because of their ease of use, applicability to wide range of patients, and ability to provide patient-personalized training [1–3]. Additional reported benefits of virtual reality systems for both patients and health providers include increased therapy efficiency and a high level of attention in patients during training [4].

One of the main struggles therapists encounter is keeping patients motivated throughout conventional training sessions. The Yerkes-Dodson Law describes the relationship between arousal or motivation and performance [5]. At first, an increase in arousal and motivation leads to an increase in performance. But once a certain point is reached, this point can vary based on many factors including the task, the participant, and the context, the relationship becomes inverse and increases in arousal caused decreases in performance. In line with these ideas, previous research has shown that increased performance leads to greater improvement in patients after stroke up to a certain point. Virtual reality-based systems allow manipulation of arousal through training settings to ensure that peak performance is maintained for as large a portion of the therapy time as possible [6].

Laver et al. systematically evaluated the literature regarding the efficacy of virtual reality-based training in stroke rehabilitation in 2011 and in its updates in 2015 and 2017 [3, 7, 8]. Their current meta-analysis of 22 trials including 1038 patients after stroke that focused on upper limb function did not reveal a statistically significant difference between VR-based training and conventional therapy (0.07 standard deviation higher in virtual reality-based compared to conventional therapy. Furthermore, the authors rated the quality of evidence as low, based on the GRADE system. However, for ADL function the experimental groups showed a 0.25 higher standard deviation than the conventional therapy groups based on ten studies, including 466 patients after a stroke with moderate quality of evidence.

Only 10% of the included studies included more than 50 participants, with mean ages between 46 to 76 years. However, due to the different systems used no conclusion could be drawn regarding grip strength, dosage, type or program of the virtual reality-based training. Furthermore, the authors pointed out the low sample sizes and the low methodological quality of the reported trials. In their recommendations for further research, the authors encouraged researchers and clinicians again to conduct larger trials and to increase the detail in reporting to enable more firm conclusions.

YouGrabber (now renamed Bi-Manu Trainer), a game-based virtual reality system designed for upper-limb rehabilitation, has been shown to be effective in children with cerebral palsy. A 2-subject feasibility study indicated that the findings might extend to chronic stroke patients [9, 10]. Both male subjects, who were trained three years after insult onset, showed increases in scores for the bimanual activities of daily living focused Chedoke McMaster Arm and Hand Activity Inventory (CAHAI) that persisted at the final follow-up, and corresponding cortical changes measured with fMRI.

Based on these findings the present multicenter parallel group randomized single-blinded trial aimed to investigate the efficacy of a virtual reality-based training with the YouGrabber training device (now renamed Bi-Manu Trainer) compared to conventional therapy. The study was designed to test the hypothesis that patients in the chronic stage after stroke in the virtual reality-based training group will show no higher post-intervention performance in the Box and Block Test (BBT) compared to patients receiving an equal training time of physiotherapy or occupational therapy.

For comparison with published and ongoing international studies we selected the Box and Block Test as the primary outcome measure and the CAHAI as the secondary outcome measure.

## Methods and materials

### Study design

This prospective, multicenter, single-blinded, parallel-group randomized trial was conducted in the outpatient departments of three rehabilitation hospitals in the German and French speaking parts of Switzerland: University hospital Inselspital Bern, Buergerspital Solothurn, and Reha Rheinfelden. In the study plan, each hospital was responsible for the recruitment, assessment, and therapy of 20 patients: 10 patients for the experimental group (EG) and 10 for the control group (CG), respectively.

**Fig 1:**
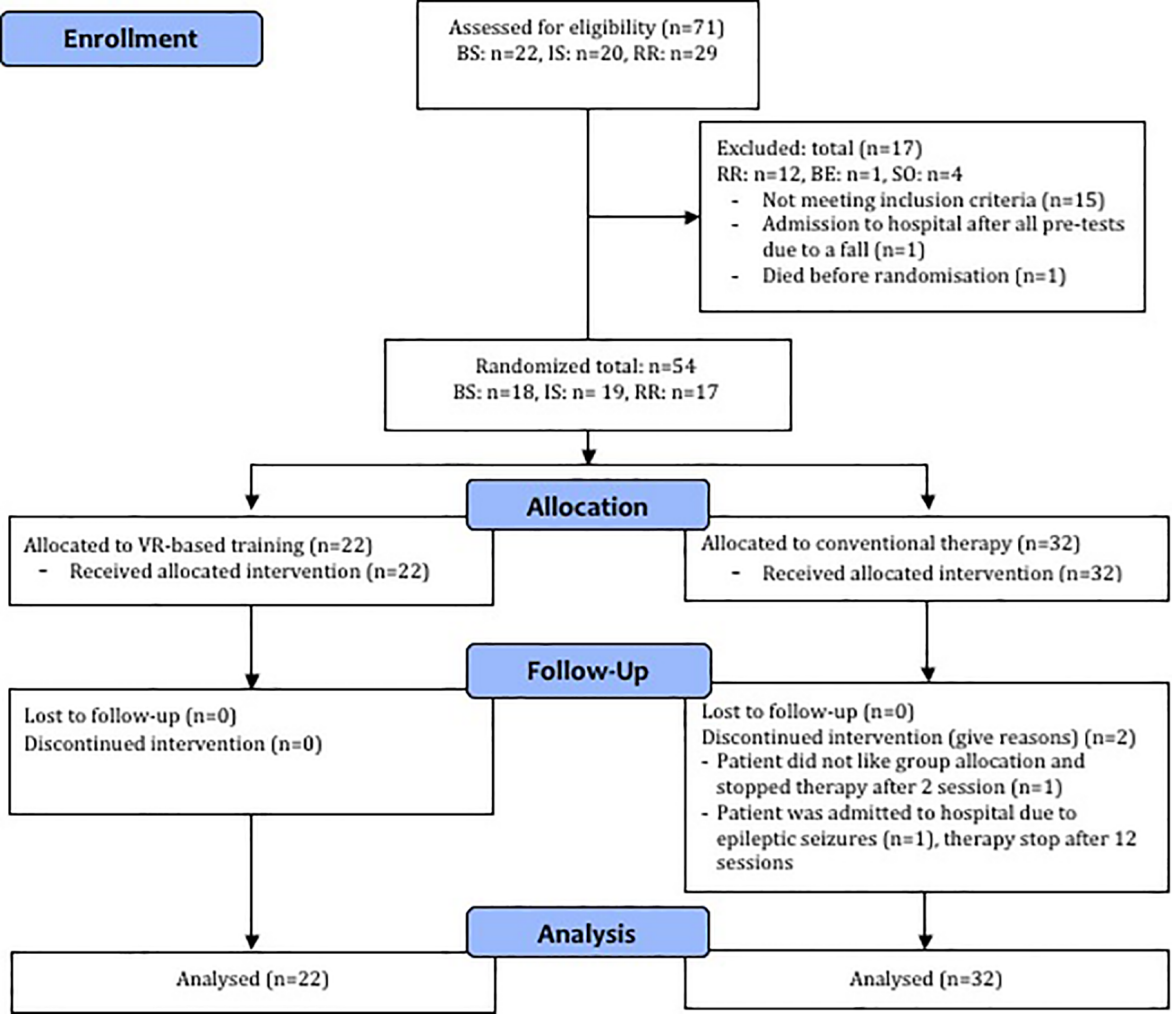
Patient flow chart. BS = Buergerspital Solothurn, IS = Inselspital Bern, Reha Rheinfelden Measurement sessions: twice within one to two weeks before intervention start (BL, T0), once after eight (T1) and after 16 (T2) intervention sessions, and after a two months follow-up period (FU).

More details regarding the study methodology can be found in the study flow chart in Fig 1 and the previously published study protocol strictly followed by each center (http://trialsjournal.biomedcentral.com/articles/10.1186/1745-6215-15-350) [11]. Ethics approval was warranted by the ethics committee of the Canton Aargau (2012/065) and the Canton Berne (220/12). The study was registered with ClinicalTrials.gov: NCT01774669 before the start of patient recruitment.

### Participants

In order to meet the inclusion criteria, each patient had to be at least six months after his or her first-ever stroke (ischemic or hemorrhagic) with a persistent motor deficit of the arm and hand, indicated by a Chedoke-McMaster Stroke Assessment (CMSA) score of three or greater on the arm subscale and two or greater on the hand subscale. If one of the CMSA subscales scored seven, the difference to the other subscale had to be at least two. Patients had to be able to sit in a normal chair without armrests or backrest support and to score at least one on the Box and Block Test, which was the primary outcome measure. Patients were excluded from the study if they had previous or current functional deficits of the arm and hand motor function not due to stroke, severe cognitive deficits indicated by a Mini-Mental State Examination score of 20 or lower, severe visual disorders, or a history of epileptic seizures triggered by visual stimuli within the past six months.

Patients were informed about the study in oral and written form and gave written informed consent before data collection began.

Furthermore, for descriptive purposes patients were evaluated with the Extended Barthel Index (EBI) [12], the Edinburgh Handedness Inventory (EHI) [13], the Mini Mental State Examination (MMSE) [14], and with the Line Bisection Test (LBS) [15]. Table 1 provides an overview of all outcome measures and measurement sessions.

**Table 1:** Outcome measures and measurement sessions.

Assessment	Abbreviation	Outcome	Measurement sessions				
			BL	T0	T1	T2	FU
**Primary outcome**							
Box and Block Test	BBT	Hand dexterity	X	X	X	X	X
**Secondary outcomes**							
Chedoke McMaster Arm and Hand Activity Inventory	CAHAI-13	Activity (ADL)	X	X	X	X	X
Stroke Impact Scale	SIS	Impact of stroke on ADL, mobility, emotion, memory, strength, communication	X	X	X	X	X
**Outcomes for descriptive purposes**							
Extended Barthel Index	EBI	Independence in ADL	X				
Chedoke-McMaster Stroke Assessment	CMSA	Motor impairment	X				
Edinburgh Handedness Inventory	EHI	Handedness	X				
Mini Mental State Examination	MMSE	Cognitive screening	X				
Line Bisection Test	LBT	Neglect	X				

ADL = Activities of daily living; BL = Baseline, FU = Follow-up 2 months after study treatment finalization; T0 = Pre-intervention; T1 = after eight intervention sessions; T2 = posttest after 16 intervention sessions.

### Randomization and masking

If a patient met all eligibility criteria, he/she was randomly assigned to either the experimental group (EG) or the control group (CG) after the second baseline measurement session.

Group allocation (1:1 ratio) was based on one computer-generated randomization list for all centers. The randomization list was created on blocks of 10 and was generated by a researcher not involved in the study (MATLAB release 2007b; MathWorks, Natick, MA, USA).

The randomization list was stored at one clinic’s pharmacy only to avoid disclosing the group allocation. The treating therapist of the respective clinic called the pharmacy before the first therapy or training appointment of the respective patient to obtain the group allocation. Only study therapists, who were specifically trained in the study methodology, were allowed to call, which was verified by the pharmacist on duty. Group allocation was not recorded on any assessment document. Group allocation was only noticeable based on the selected training or therapy documentation sheets that were kept locked and separated from the assessment documents. A row of measures was implemented to keep group allocation concealed from the blinded assessors until the last follow-up measurement session of the last patient. Two patients disclosed their group allocation during the third out of five measurement sessions. In these cases, the measurement sessions three to five were video recorded to ensure objective and unbiased assessment scoring.

### Procedures

Patients were assessed at five measurement sessions: twice within one to two weeks before intervention start (BL, T0), once after eight (T1) and after 16 (T2) intervention sessions, and after a two-month follow-up period (FU). For statistical analyses, average scores of BL and T0 were used as one pre-intervention score = (BL+T0)/2.

The intervention consisted of four 45-minute training sessions per week over a four-week period for patients in both groups. The EG underwent a virtual reality-based training for all 16 sessions and the CG underwent conventional physiotherapy or occupational therapy. The therapy and virtual reality-based training are described using the Template for Intervention Description and Replication (TIDieR) in Table 2 [16].

**Table 2:** Study intervention description based on the TIDieR checklist [16].

Item		Experimental group	Control group
1	Brief name	Virtual reality-based training system	Conventional therapy
2	Why	Both interventions were compared directly in chronic stroke patients for two reasons:	
		1. One-to-one therapy sessions in an adequate amount are limited by health insurance company restrictions.	
		2. If virtual reality-based technology is used, and YG in particular, patients and therapists wanted to know if the treatment effect is the same. If yes, YG could be used to increase the amount of training time with the technology, or it could be recommended as group- or home-based virtual reality training, which would not be the case if YG performed worse.	
3	What: materials	EG patients were sitting or standing in front of the virtual reality training system. They wore hand gloves with attached sensors to measure finger movements of the thumb, index finger, middle finger, wrist (bending, extending) and lower upper limb (pronation, supination). Movements were displayed on the screen in real time.	No restrictions were placed on the material used (for example, ADL material, reaching and grasping material). Use of additional electrical or mechanical therapy devices (for example, help arm systems, splints) were avoided.
4	What: procedures	The virtual reality-based system has a variety of training applications for different movements and at different levels of difficulty. Therapists could select one of three modes to control the on-screen finger and arm movements: (1) use of the real arm and/or hand movements, (2) mirroring of the real movements of one arm and/or hand and (3) following the movements of one arm and/or hand. The distribution and speed of the appearing objects were attuned. Furthermore, patients’ movements could be amplified or modulated in the virtual environment to force decreases or increases in training difficulty [17]. After the second virtual reality-based training session, patients had tested all training applications and all three modes of finger and/or hand movements. In the remaining 14 sessions, therapists selected at least 3 training applications for each training session and 2 different movement modes with settings adapted to each patient’s needs.	The therapy content focused on a task-related upper-limb treatment in a sitting or standing position. Several manual techniques, therapy materials and objects of ADL were performed [18, 19]. Three main aspects were considered during therapy: (1) neuromuscular interventions (NDT)–about 75% of the therapy content, (2) body structural interventions (BSI)–about 20% of the therapy content, (3) perceptual and sensory interventions (PSI)–about 5% of the therapy content. (1) NDT included neurodevelopmental/motor learning treatment focusing on postural control (5%), fine and gross motor skills (65%), and coordination (30%). (2) BSI included stretching (5%), passive/assistive mobilization of body structures and joints (45%), and training for specific muscles or muscles groups in an assistive, active or resistance mode (50%). (3) SI included proprioception (25%) and haptic perception (75%) exercises.
5	Who provides	Both study interventions were provided by experienced physiotherapists or occupational therapists, who had at least 2 years of professional experience in the field of neurorehabilitation.	
6	How	Both study interventions were conducted individually in one-to-one sessions.	
7	Where	Both study interventions took place in the physiotherapy or occupational therapy department of each participating center.	
8	When and how much	During the 4-week intervention program, patients in both study groups (EG, CG) received the same amount of 16 sessions lasting 45 minutes each. Therapist and patient contact time varied between 25 to 60minutes (average 45·3min) for both groups including greeting, organization of next appointment, short clinical examination, changes since last appointment, training or therapy itself, and farewell. Patients in the EG performed between 267 to 4283 grasps if the right paretic hand/arm and 102 to 5077 grasps if the left hand/arm was paretic over the 16 training sessions.	
9	Tailoring	Training and therapy content was tailored to each patient’s preferences, the agreed movement aims and the motor function level of each patient.	
10	Modifications	No modification occurred during the course of the study.	
11	How well	All 22 patients (100%) in the virtual reality-based training group and 30 (93.8%) patients in the conventional therapy group completed the training. That was evaluated by the training and therapy documentation forms for both groups that were filled in during each training session.	
12			

ADL, Activities of daily living; CG, Control group; EG, Experimental group; TIDieR, Template for Intervention Description and Replication checklist and guide; VR, Virtual reality; YG, YouGrabber (now renamed Bi-Manu Trainer).

If patients received any kind of additional therapy before trial participation, it was reduced or suspended for the course of the study if the patient agreed. If the additional therapy had to be continued or could not be reduced, it was ensured that its focus was on lower extremity treatments.

### Outcomes

Table 1 lists the primary outcome measure and all secondary outcome measures for each measurement session. Adverse events were registered and transferred to the responsible ethics committee if the applicable criteria for transfer were met. A detailed description of all outcome measures can be found in the published study protocol (https://trialsjournal.biomedcentral.com/articles/10.1186/1745-6215-15-350).

Changes in hand dexterity between T0 and T2 were measured with the BBT, which was described by Mathiowetz et al. in 1985 [20]. Patients were asked to grasp small wooden cubes and move them from one side of the box to the other as fast as possible within 60 seconds. The BBT provides normative data for healthy individuals in age groups ranging from 20 years to older than 75. A change of five or six cubes before and after an intervention seems to be the smallest real difference [21].

The CAHAI-13 was developed by Barreca et al. in 2004 [22–25]. It contains 13 bimanually performed real-life items. Scores represent the patient’s relative ability to independently perform stabilisation or manipulation in ADL with the impaired upper limb. A score of one represents total dependence on another person, and a score of seven indicates patient independence without time or safety concerns or necessary splints or devices.

The SIS is a questionnaire comprising questions regarding the impact of stroke on physical function, emotion, memory, communication and social participation. The SIS was developed by Duncan and colleagues and has been modified in recent years [26–28]. The current version, 3.0, consists of eight subscales (strength, hand function, mobility, ADL, emotion, memory, communication and participation) administered in a one-to-one interview. Patients can rate the level of their stroke’s impact on a 5-point Likert scale. The higher the score, the less affected the patient perceives his or her current status to be.

The Extended Barthel Index was used for patient evaluation of independence in ADL [12]. The EBI comprises 16 items on mobility, ADL and cognitive function. Scoring ranges from zero to four with four indicating the highest level of independence.

The CMSA was developed by Gowland et al. in 1995 for the evaluation of physical impairment and activity level of stroke patients [29]. We used the impairment subscales for hand and arm function that was scored on a seven-point scale (1 = hypoactive or absent muscle reflexes, 7 = no functional impairment detectable anymore, prestroke status) according to seven stages of motor recovery [30]. Additionally, the subscale of shoulder pain of the affected body side was administered on the same seven-point scale.

The Edinburgh Handedness Inventory was used to assess hand laterality [13]. The questionnaire included 12 daily activities were participants had to determine their preferred hand (right/left).

For cognitive screening the Mini-Mental State Examination (MMSE) was conducted. The MMSE comprised 30 items and patients could achieve zero to 30 points (indicating the highest scoring) [14].

The Line bisection test (LBT) is a paper-and-pencil test used to evaluate the presence of unilateral spatial neglect [15]. Patients were asked to mark the centre of 18 drawn lines on paper with a pencil.

### Statistical analyses

The sample size was calculated based on the basis of an earlier efficacy study, in which the virtual reality-based system was tested in children with cerebral palsy [9]. A power analysis and a sample size calculation for the present study were performed using G*Power software version 3.1.5 [31]. In the cited study of children, the BBT (primary outcome measure) showed an effect size of Cohen’ s d = 0.98. Assuming a similar effect size for adult stroke patients, a total of 46 patients (23 per group) had to be included: two-tailed test, power = 0.9, significance level α = 0.05. Assuming a dropout rate of 20%, we thus planned to recruit a total of 60 patients across the participating centers.

All statistical analyses were conducted using the Statistical Package for Social Sciences (SPSS) version 23.0 (IBM, 290 Armonk, New York, USA) with a two-sided significance level of p≤0.05 as an intention-to-treat analysis.

Variables were checked for normal distribution with the Shapiro-Wilk test.

1. Due to observable mean differences at baseline we checked for baseline significant differences for age, time since stroke, BBT, CAHAI, and SIS subscale hand function (subscale 7). Due to non-normal distribution, we used the Mann-Whitney U-Test that did not reveal significant differences between EG and CG at BL.
2. Due to non-normal distribution of primary and secondary outcome measures we calculated the differences between each measurement session. Again, the differences were tested for normal distribution, which was not the case. Subsequently, the differences were tested using the Friedman test for repeated measures to determine changes over time for both groups. The Mann-Whitney U-Test was used to test for group differences of the changes for each measurement session.
3. All outcome analyses were intention-to-treat analyses with missing values replaced with two methods: carrying the last observable value forward or backward and by adding or subtracting the mean change of the group [32, 33]. This sensitivity analysis did not materially change the results. For the primary outcome analysis, the differences of the BBT scores of the paretic side for each measurement session were the dependent variables. For the secondary outcome analyses, dependent variables were the difference scores of the CAHAI, the SIS subscales 1, 5, 6, 7, 9, the SIS mobility index, and the BBT scores of the non-paretic side. To correct the p-value for multiple comparisons the Bonferroni adjustment (p = 0·05/k) was used, where k represents the number of tests for significance (k = 3) [34].
4. No post-hoc power analysis was conducted due to the lack of a group interaction effect. Nevertheless, we calculated the effect for the four-week training intervention for each group separately and the standardized mean difference between groups with the following formula: Kazis’ effect size = (pre-intervention score-post-intervention score)/standard deviation of pre-intervention score [35].

## Results

The study was conducted between December 1, 2012, and February 15, 2016 including the last follow-up assessment. In total, with a recruitment rate of 1.3, 54 patients were included, of whom 22 patients received virtual reality-based training (40.7%) and 32 (59.3%) patients received conventional therapy. All 22 patients (100%) in the virtual reality-based training group and 30 (93.8%) patients in the conventional therapy group completed the training.

Table 3 provides an overview of all patient baseline characteristics and Fig 1 shows the study patient flow chart. There were no baseline characteristic differences except for the SIS mobility index with p = 0.05 (please see Table 4).

**Table 3:** Patients’ baseline characteristics for personal, diagnosis- and screening-related information.

	Virtual reality-based training (n = 22) (mean ± SD, range)	Conventional therapy (n = 32) (mean ± SD, range)
Age (years)	61.3 ± 13.4 (22.9–81.0)	61.2 ± 11.2 (20.0–78.3)
Gender (female/male)	6 / 16	9 / 23
Marital status (married / living alone)	15 / 7	21 / 10*
Time since stroke (years)	2.4 ± 2.4 (0.4–9.5)	3.6 ± 3.7 (0.45–13.7)
Time of additional PT and/or OT (min/week)	67.1 ± 44.5 (0–180)	83.3 ± 56.0 (0–210)
Extended Barthel Index (max. 64)	60.4 ± 5.6 (41–64)	59.5 ± 6.7 (30–64)
Mini-Mental State Examination (max. 30)	28.6 ± 1.0 (27–30)	28.4 ± 2.0 (23–30)
Chedoke-McMaster Stroke Assessment		
Subscale: Shoulder pain (max. 7)	5.2 ± 1.1 (4–7)	5.4 ± 1.4 (2–7)
Subscale: Arm function (max. 7)	4.0 ± 1.0 (3–7)	4.0 ± 1.0 (3–6)
Subscale: Hand function (max. 7)	4.3 ± 0.9 (3–6)	4.2 ± 1.4 (2–7)
Stroke (ischemic/hemorraghic)	18 / 4	25 / 7
Dominant side paretic (n)		
Right hand dom.+par.	13	14
Right hand dom.+left hand par.	8	15
Left hand dom.+par.	0	1
Left hand dom.+right hand par.	1	2
Experience with		
Working with a PC (yes/no)	21*/0	25/6*
Virtual reality (yes/no)	21*/0	17/14*
PC games (yes/no)	21*/0	16/15*

The Mann-Whitney U-Test was used to determine significant differences between EG and CG at BL. However, there were no baseline for the parameters displayed in Table 3.

* = data of one participant missing, ADL = Activities of daily living, PT = physiotherapy, OT = occupational therapy, dom. = dominant, par. = paretic, VR = virtual reality. Numbers in brackets represent median and range (rounded).

**Table 4:** Details of primary and secondary outcomes.

Outcome measure	YouGrabber Training n = 22 Mean changes over time compared to pre-intervention score		Conventional therapy n = 32 Mean changes over time compared to pre-intervention score		Between group differences for all ME	
	Median (IQR)	p-value*	Median (IQR)	p-value*	Z-value	p-value
**Primary outcome**						
***Box and Block Test paretic side***						
Pre-intervention (T0)	19.3 (11.9 to 36·0)	—	17.8 (4.3 to 28·0)	—	-0.863	0.388
After 8 trainings (T1)	22.5 (13.8 to 37.8)	0.006*	21.0 (6.3 to 33.8)	0.002*	-0.317	0.751
After 16 trainings (T2)	22.0 (15.3 to 35.8)	0.02	20.5 (5.5 to 34.5)	0.003*	-0.203	0.839
After 2 months (FU)	19.5 (15.5 to 39.3)	0.069	21.5 (7.3 to 36.3)	0.001*	-0.951	0.341
**Secondary outcomes**						
***Chedoke McMaster Arm and Hand Activity Inventory (13–91)***						
Pre-intervention (T0)	73.8 (47.0 to 83.6)	—	72.0 (44.6 to 84.4)	—	-0.297	0.771
After 8 trainings (T1)	75.0 (59.8 to 86.8)	0.001*	77.5 (43.8 to 87.0)	0.002*	-1.375	0.169
After 16 trainings (T2)	77.0 (61.2 to 85.0)	0.01*	72.0 (47.3 to 89.0)	≤0.001*	-0.864	0.387
After 2 months (FU)	77.0 (60.0 to 89.0)	0.001*	77.0 (45.3 to 87.0)	0.002*	-0.546	0.585
***Stroke Impact Scale*: *subscale 1 strength (0–100)***						
Pre-intervention (T0)	60.9 (52.3 to 72.7)	—	59.4 (43.8 to 68.8)	—	-0.371	0.711
After 8 trainings (T1)	68.8 (54.7 to 71.9)	0.037	62.5 (50.0 to 73.4)	0.713	-1.659	0.097
After 16 trainings (T2)	68.8 (50.0 to 78.1)	0.001*	62.5 (50.0 to 73.4)	0.159	-2.189	0.290
After 2 months (FU)	68.8 (56.3 to 75.0)	0.002*	65.6 (50.0 to 75.0)	0.48	-1.904	0.057
***Stroke Impact Scale*: *subscale 5 activities of daily living (0–100)***						
Pre-intervention (T0)	83.6 (65.4 to 90.9)	—	73.4 (65.4 to 83.3)	—	-1.294	0.196
After 8 trainings (T1)	86.5 (69.8 to 97.2)	0.001*	77.1 (70.8–86.5)	0.15	-1.55	0.121
After 16 trainings (T2)	89.6 (70.3 to 97.9)	0.001*	81.3 (68.8–87.0)	0.004	-1.340	0.180
After 2 months (FU)	90.2 (78.1 to 95.8)	≤0.001*	83.3 (64.6–87.5)	0.052	-1.532	0.125
***Stroke Impact Scale*: *subscale 6 mobility (0–100)***						
Pre-intervention (T0)	90.0 (74.7 to 99.1)	—	86.9 (76.6 to 94.4)	—	-1.235	0.217
After 8 trainings (T1)	93.8 (81.8 to 100.0)	0.015	77.1 (70.8 to 86.5)	0.191	-0.65	0.516
After 16 trainings (T2)	92.5 (83.8 to 100.0)	0.091	92.5 (78.1 to 96.9)	0.015	-0.265	0.791
After 2 months (FU)	95.0 (81.9 to 100.0)	0.243	91.3 (78.1 to 97.5)	0.302	-0.204	0.839
***Stroke Impact Scale*: *subscale 7 hand function (0–100)***						
Pre-intervention (T0)	65.0 (37.5 to 81.3)	—	43.8 (20.6 to 74.4)	—	-1.648	0.099
After 8 trainings (T1)	75.0 (45.0 to 86.3)	0.001*	62.5 (25.0 to 80.0)	0.004	-0.601	0.548
After 16 trainings (T2)	77.5 (55.0 to 86.3)	0.004*	62.5 (21.3 to 85.0)	≤0.001	-0.574	0.566
After 2 months (FU)	72.5 (62.5 to 90.0)	0.011*	72.5 (25.0 to 90.0)	0.001	-0.538	0.591
***Stroke Impact Scale*: *subscale 9 stroke recovery (0–100)***						
Pre-intervention (T0)	54.5 (44.4 to 72.8)	—	61.3 (47.0 to 70.0)	—	-0.890	0.374
After 8 trainings (T1)	60.0 (50.0 to 77.3)	0.003*	60.0 (55.0 to 73.8)	0.277	-1.107	0.268
After 16 trainings (T2)	64.0 (53.8 to 76.3)	0.001*	64.0 (50.0 to 77.3)	0.033	-1.603	0.109
After 2 months (FU)	69.0 (48.8 to 75.0)	0.012*	70.0 (50.0 to 78.0)	0.288	-0.411	0.681
***Stroke Impact Scale*: *mobility index (0–5)***						
Pre-intervention (T0)	4.4 (3.7 to 4.5)	—	3.8 (3.5 to 4.3)	—	-1.960	**0.050**
After 8 trainings (T1)	4.6 (4.0 to 5.0)	0.002*	4.0 (3.5 to 4.5)	0.006	-0.238	0.812
After 16 trainings (T2)	4.6 (4.0 to 5.0)	0.023	4.0 (3.5 to 4.5)	0.003	-0.106	0.916
After 2 months (FU)	4.6 (4.0 to 5.0)	0.006*	4.0 (3.5 to 4.5)	≤0.001	-0.108	0.914
***BBT non-paretic side***						
Pre-intervention (T0)	54.0 (44.8 to 59.9)	—	49.8 (43.5 to 56.6)	—	-1.057	0.291
After 8 trainings (T1)	58.0 (45.8 to 65.0)	0.001*	50.5 (45.0 to 59.5)	0.033	-1.216	0.224
After 16 trainings (T2)	57.5 (46.0 to 65.0)	0.008*	53.0 (46.3 to 59.8)	0.001	-0.273	0.785
After 2 months (FU)	59.0 (45.8 to 64.5)	≤0.001*	54.5 (46.3 to 63.8)	0.001	-0.273	0.785

The Friedman test for repeated measures was used to determine changes over time for both groups. The Mann-Whitney U-Test was used to test for group differences of the changes for each measurement session. A significant difference was found for the SIS mobility index at pre-intervention. The p-value is marked in bold.

BBT = Box and Block Text, CAHAI = Chedoke McMaster Arm and Hand Activity Inventory, FU = Follow-up, SIS = Stroke Impact Scale, SS = subscale

* significant p-Level after Bonferroni adjustment (p = 0.05/k)

k represents number of tests for significance (k = 3).

**Fig 2:**
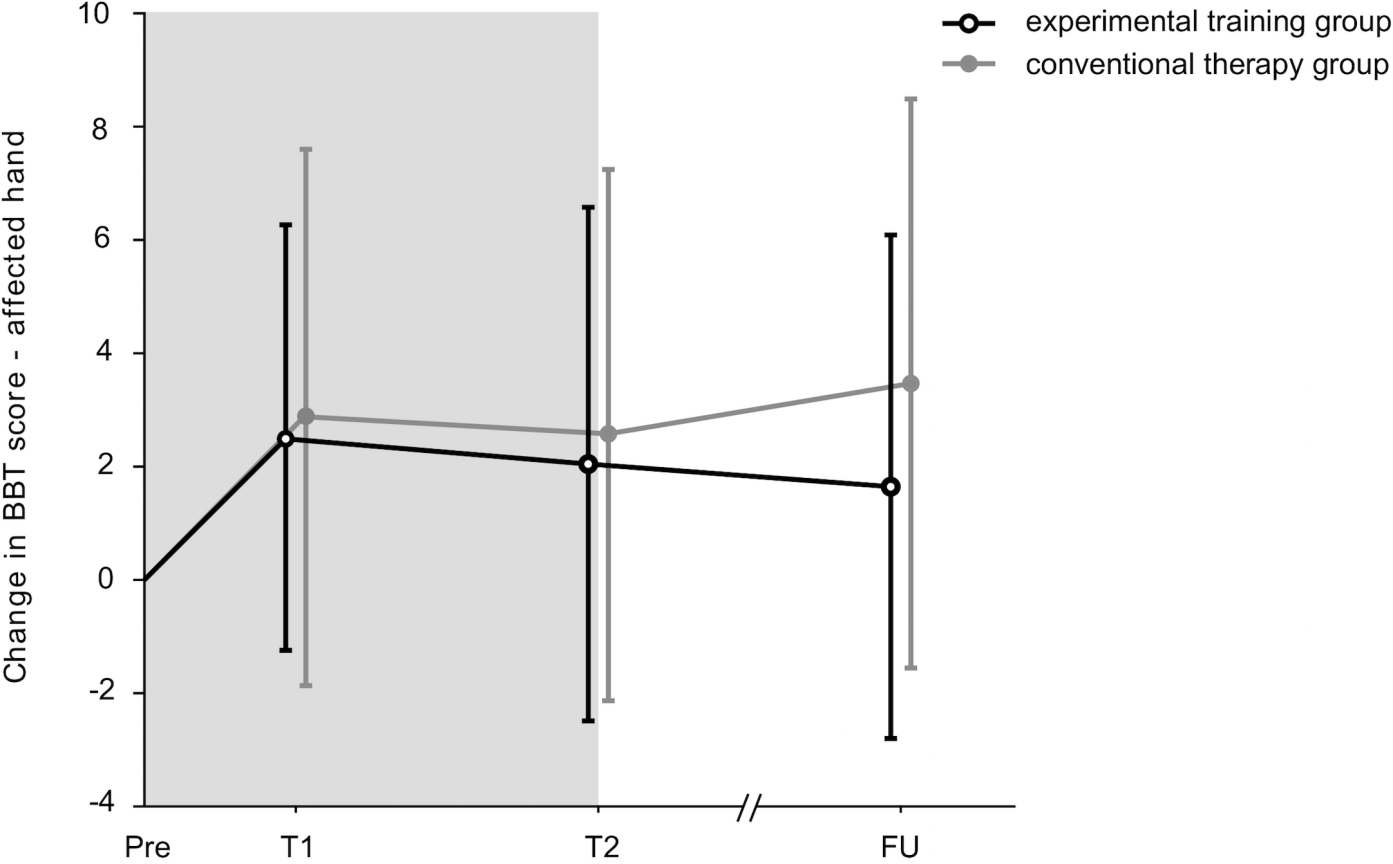
Change in Box und Block Test: paretic hand. Pre = Pre-intervention, T1 = after 8 training sessions, T2 = after 16 training sessions, FU = follow-up after two months.

**Fig 3:**
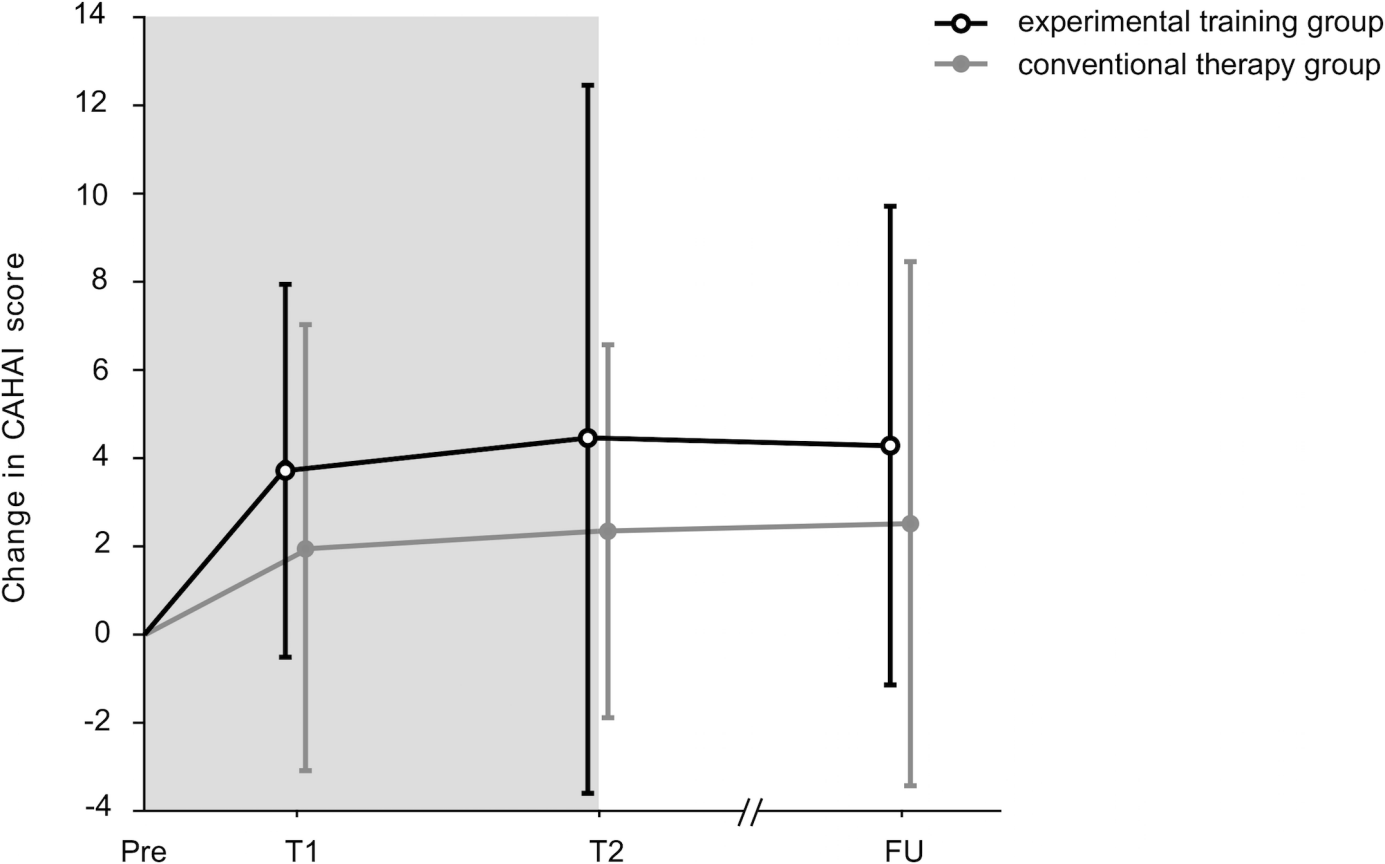
Change in Chedoke McMaster Arm and Hand Activity Inventory. Pre = Pre-intervention, T1 = after 8 training sessions, T2 = after 16 training sessions, FU = follow-up after two months.

Figs 2 and 3 illustrate the change scores for BBT of the paretic hand and the CAHAI. All change scores of the primary and secondary outcomes are provided in Table 4. supporting information S1–S4 Figs illustrate the change scores of further SIS subscales and BBT scores for the non-paretic hand.

After the intervention period (T2), which included 16 training or therapy sessions lasting 45 minutes each, both groups showed highly significant mean differences from Pre to FU for hand dexterity assessed with the primary outcome measure BBT (mean change from Pre to FU for EG: 1.7 points, for CG: 3.5 points). A similar result was found for the secondary outcome measure bilateral arm function assessed with the CAHAI-13 (mean change from Pre to FU for EG: 5.4 points, CG: 3.1 points). Based on the findings from Chen et al. and Barreca et al., a change of 5.5 points in the BBT and of 6.3 points in the CAHAI-13 would have been necessary for a clinical relevant change [21, 24]. However, for both outcomes no between-group differences were detected.

In the study protocol no subgroup analyses were pre-specified. However, two non-significant trends based on the median score of the BBT and the CAHAI became obvious and were further investigated. In subgroup analyses based on (1) the median value for BBT (18 blocks) and (2) the CAHAI (score of 72) no differences could be detected (p>0.121).

1. For patients, who scored higher or equal as the median value of 18 in the BBT at pre-intervention (EG = 12, CG = 16) there was an improving trend to T2 (p = 0.08) in favor of the experimental group. On average, patients in the experimental group improved from 10.9 (±5.0) to 13.7 (±5.8) at T2 (mean ± SD), whereas patients in the control group changed from 7.4 (±6.1) to 9.0 (±8.0) at T2 (mean ± SD).
2. For patients, who scored less or equal as the median value 72 in the CAHAI at pre-intervention (EG = 11, CG = 16) there was an improving trend to T2 (p = 0.07) in favor of the experimental group. On average, patients in the experimental group progressed from CAHAI a score of 51.6 (±12.2) to 63.3 (±14.3) at T2 (mean ± SD), whereas patients in the control group changed from a CAHAI score of 44.5 (±16.2) to 47.7 (±16.8) at T2 (mean ± SD).

Scoring of both groups in the SIS changed significantly in subscales hand function, activities of daily living, and mobility index, but not for subscale mobility. Interestingly, for subscale strength both groups showed large self-perceived increases with a trend in favor of the experimental group (p = 0.057), who improved their mean value from pre-intervention to FU by about eight points. By comparison, the control group improved their mean value by almost three points (see Table 3 and supporting information S1 to S4 Figs).

Effect sizes were reported in the supporting information S1 Table for each group separately. They range between 0.13 and 0.52 for BBT, CAHAI, and SIS upper limb related subscales, e.g. strength, ADL, and hand function, excluding subscale mobility.

For the whole course of both interventions no adverse events in relation to study participation were reported. Before randomization, three patients expected adverse events due to different reasons not related to the study and were excluded—please see the patient flow chart for details in Fig 1.

## Discussion

The present multicenter parallel group randomized controlled trial aimed to investigate the efficacy of a virtual reality-based training with YouGrabber compared to conventional therapy as stand-alone therapy in patients in the chronic stage after stroke. As hypothesized, both patient groups significantly improved their performance in primary and secondary outcomes but did not show between-group differences after eight or 16 training sessions or after the two-month follow-up. These results are in line with recent publications on VR-based training from Brunner et al. and the systematic review update from Laver et al. [8, 36]. However, three non-significant trends should be further discussed: (1) subjectively perceived improved strength trend in the virtual reality-based training group compared to the CG, (2) the main scoring increase over the first eight compared to the second eight training sessions, and (3) the increased scoring in the BBT of the non-affected upper limb.

### Comparison with other studies

Repetition of movement is one of the fundamental principles for motor re-learning and facilitating brain plasticity to improve motor function [37]. In our study, the differences in the subjectively improved strength measured with the SIS could be explained by the very high numbers of repetitions of arm and finger movements preformed during the YG games. The training in EG was very much focused on active finger, hand and arm movements that could lead up to 5000 grasp movements over 16 training sessions. However, only patients in the CG performed a resistance training with heavy objects, e.g. weights but with a lesser amount of repetitions.

The main scoring increase occurred within the first eight training sessions that were scheduled during the first two weeks of the intervention. During the second two intervention weeks, scores further increased albeit to a lesser degree or they remained on the same level. The Yerkes-Dodson Law, first explained by Yerkes and Dodson in 1908, is a dose-response framework that describes a relationship between arousal or motivation and performance and might help to explain that phenomenon [5]. It indicates that a low level of task difficulties elicits linear responses. Reaching a higher level of difficulty, the relationship becomes inverse and increases in arousal could cause decreases in performance. From our patients, we know that they enjoyed playing the virtual reality-based games and always reached for a higher score or a faster performance [38]. The intensive training could have reached a point when a higher intensity is necessary to push the functional improvements and patient motivation further, e.g. longer than 45 minutes per day or more than four times per week.

It is worth mentioning that patients in both groups scored approximately 20 points below the average score in the BBT with their non-paretic upper limb compared to healthy individuals of the same mean age. Mathiowetz et al. suggested a norm score above 70 for healthy women and men for the left and right side [20]. Patients in both groups improved their BBT scoring with 4.6 (EG) and 4.4 (CG) from baseline to follow-up. That distinct scoring change of the non-paretic upper limb over the four measurement sessions is somewhat surprising. One would assume that patients 2.5 to 3.5 years after stroke would use the non-paretic upper limb more throughout the day to compensate for the reduced motor function of the paretic side and would have developed even more hand and arm dexterity over time compared to healthy individuals. However, it could be hypothesized that the non-paretic hand/arm compensated for the non-use of the paretic hand/arm. Furthermore, the reduced overall activity and motor function after stroke and intensive carer assistance could have led to a more sedentary lifestyle and a learned non-use for the non-paretic side as well. A BBT performance difference of the paretic and non-paretic arm depending on side of brain lesion as suggested by Steward et al. could not be detected [39].

Overall, our results are in line with the previous publications comparing virtual reality-based training using a commercial consumer virtual reality training systems, e.g. Nintendo Wii, XBox, Playstation with conventional therapy including patients in a chronic stage after stroke [40–42]. One difference in our study was that we used a system that was specifically developed for patients with sensorimotor impairments. The system offers different display modes of the arms (a) real left/right hands control their virtual counterparts, (b) virtual mirror therapy, in which one real hand controls both virtual arms or the contralateral virtual hand in a mirrored fashion, or (c) virtual following that is the same as for virtual mirror therapy, but without mirroring [43].

Our results continue the work from Saposnik et al., who included 121 light to moderately impaired inpatients in a subacute stage after stroke [44]. They used a commercial virtual reality-based system for a two-week training program including ten 60-minute sessions as an adjunct to the multidisciplinary program and found significant improvements over time for both groups but no between-group differences.

Furthermore, our results are in line with findings from a recent systematic review on virtual reality-based training in stroke rehabilitation from Aminov et al. [45]. Researchers reported small to moderate effect sizes in favor of virtual reality-based training ideally performed with a purposed-designed virtual reality design system similar to the system used in our multi-center study. Supporting our study design the authors of the systematic review and meta-analysis reported higher effects in favor of virtual reality-based training for patients three or more months post stroke, with more than 15 training sessions in total including more than three training sessions per week adding up to more than 100 minutes per week. We also included outcome measures to evaluate all three main categories of the International Classification of Function: body structure and function, activity, and participation.

Future research directions should consider the potential of virtual reality-based training system to increase the efficiency of training in terms of human resources. Currently, the number of available therapists is not sufficient compared to the increasing number of patients. To supply the required dose of therapy to the number of patients after stroke experiencing motor deficits, it would be interesting if our results could be replicated with virtual reality-based group training sessions compared to individually supervised trainings. With virtual reality, most of the time of the therapy session could be automated and therefore could be completed without the constant supervision of a therapist. Furthermore, a system could even be installed in the patient’s home and the setup instructions given remotely, removing the burden of clinic visits.

### Limitations and strengths

One limitation of our study is the number of patients per group. Despite the well-prepared randomization scheme the imbalances occurred by chance. Patients were randomized at study entry but held confidential until they passed both baseline assessments (BL, T0). However, it happened that patients did not pass the baseline assessments or had to be excluded for several reasons, which led to an uneven number of patients per group and could have therefore led to an under- or overestimation of the effect of the virtual reality-based training.

A common problem in RCTs investigating therapies or training procedures is in blinding the patients regarding their group allocation. In our study, only one patient withdrew study participation after being randomized into the control group. All other patients stated that they were happy with their group allocation and showed high compliance. The study had a very low dropout rate of 1.6%.

Our sample size calculation was based on a previous study that used the same VR-based training system and included children with cerebral palsy. Their effect size was higher compared to other VR-based training systems [8].

A remarkable strength of our study is the inclusion of moderate to severely impaired patients in both groups, who were able to move at least one wooden cube in the BBT. The adjustable virtual reality-based system was adaptable to amplify even very tiny movements and therefore enabled the patient to play or train with his/her severely impaired hand or arm. That could have led to an increased motivation and desire to move or use the paretic upper limb during the day more often.

From Kwakkel et al. and Verheyden et al. we know that recovery occurs mainly in the first six month [46, 47]. However, the majority of our patients started later than six months post stroke. Their improvement could reflect some training effect on a functional level or the use of behavioural adaptation strategies rather than by restitution of existing underlying impairments themselves.

In our study, it was an important aspect to open the black box of conventional therapy and provide the clinician with specific information on the therapy content of the CG. Therefore, we analyzed and summarized the content of the therapy down to a very detailed level based on the classification system laid out by Pollock and colleagues in their Cochrane review on interventions for improving upper limb function after stroke [48].

## Conclusions

In conclusion, with the YouGrabber (now renamed Bi-Manu-Trainer) we used a virtual reality-based training system that was specifically developed for patients with sensorimotor impairments with three different display modes of the hand and arms as a safe training option. Virtual reality-based training and conventional physiotherapy and occupational therapy did not show significant differences when applied as a supervised one-to-one training. Virtual reality-based training and conventional therapy showed differently weighted therapy contents. However, considering the increasing numbers of patients after stroke in the future and the limited personnel and financial resources, a virtual reality-based training could support the rehabilitation process by increasing training time for patients with virtual reality-based group training sessions in inpatient or outpatient settings or at the patients’ home.

## Supporting information

S1 File (PDF)

S2 File (PDF)

S1 Table Effect sizes. Effect sizes range from 0.13 to 0.52 for upper limb related subscales, e.g. strength, ADL, and hand function, excluding subscale mobility.
(DOCX)

S1 Fig Change in Stroke Impact Scale: Subscale strength. Pre = Pre-intervention, T1 = after 8 training sessions, T2 = after 16 training sessions, FU = follow-up after two months.
(TIF)

S2 Fig Change in Stroke Impact Scale: Subscale activities of daily living. Pre = Pre-intervention, T1 = after 8 training sessions, T2 = after 16 training sessions, FU = follow-up after two months.
(TIF)

S3 Fig Change in Stroke Impact Scale: Subscale stroke recovery. Pre = Pre-intervention, T1 = after 8 training sessions, T2 = after 16 training sessions, FU = follow-up after two months.
(TIF)

S4 Fig Change in Stroke Impact Scale: Mobility index. Pre = Pre-intervention, T1 = after 8 training sessions, T2 = after 16 training sessions, FU = follow-up after two months.
(TIF)
