# Supplementary material for: Effect of a four-week virtual reality-based training versus conventional therapy on upper limb motor function after stroke: A multicenter parallel group randomized trial
Source: PLoS One. 2018 Oct 24;13(10):e0204455. doi: 10.1371/journal.pone.0204455 (PMC6200191; doi:10.1371/journal.pone.0204455)
Supplement: S2 File — (PDF) [file pone.0204455.s002.pdf]

# Effectiveness of the YouGrabber system using virtual reality in stroke rehabilitation: study protocol of a single blinded, randomised controlled multi-centre trial

## 1 Project organisation

### Study leader


PD Dr. Daniel Kiper — Institute for Neuroinformatics
University of Zurich and ETH Zurich
Winterthurerstrasse 190
8057 Zürich


### Investigators and participating centres


Martin Verra — Inselspital-Stiftung Bern
Institute for Physiotherapy
3010 Bern

Dr. Jürg Bernhard — Bürgerspital Solothurn
Schöngrünstrasse 38
4500 Solothurn

Dr. Corina Schuster — Reha Rheinfelden
Salinenstrasse 98
4310 Rheinfelden


### Sponsors

Dr. K. Eng — YouRehab Ltd.
Technoparkstrasse 1
8005 Zurich

The Commission for Technology and Innovation CTI Effingerstrasse 27
3003 Bern

### List of employees for each centre:

| Inselspital Bern | Bürgerspital Solothurn | Reha Rheinfelden |
|---|---|---|
| Isabelle Lehmann | Sandra Signer | Prof. Dr. Th. Ettlin |
| Esther Marigliano | Mandy Schinke | Zorica Suica |
| Jos Nieuweuhuizen | Christine Zwicky | Michael McCaskey |
| Sandra Briner | Kathrin Hofer | |
| Irène Thaler | | |
| | Nancy Frlijc | |
| | Iris Gerber | |

## 2 Background

Around 16000 people suffer a stroke in Switzerland each year, over 1 million in the rest of Europe and similar numbers in each of North America and Asia. One third of these will have long-lasting upper limb dysfunction, requiring intensive long-term rehabilitation, resulting in the urgent situation of millions of patients without a means for accessing the intensive training of arm function required for recovery [1-3].

Therapy systems employing virtual reality (VR) technology have the potential to both improve therapy quality and reduce therapy costs by i) increasing the range of possible training tasks, thereby partly automating and quantifying therapy procedures, ii) improving patient motivation and thus increasing therapy dosage using real-time task evaluation and reward, and iii) optimally recruiting the injured neural networks [4-5]. This potential can only be fulfilled after a large scale demonstration of the therapeutic efficacy of such a therapy system. YouGrabber has been developed as the first VR-based visuo-motor training system for the rehabilitation of upper-limb function in neurological (stroke) patients that taps into all three of the above-mentioned factors. It is hypothesised that YouGrabber may optimally induce cortical plasticity and functional recovery in chronic stroke patients by recruiting the so-called Mirror Neuron System [6], a cortical network of neurons that shows activity during goal-directed hand movements as well as during observation or imagery of the same action [7-8]. There is evidence that both action observation and motor imagery [9-11] may facilitate motor activity [12] and induce cortical plasticity [13].

Many pilot studies have been conducted on VR in therapy, yet a Cochrane review of VR in upper limb stroke therapy found that there is so far insufficient evidence to decide on the efficacy of VR [14]. This is due mainly to the heterogeneous VR technologies, methods of application, methods of assessment and inconsistent study designs making it very difficult to draw statistically significant conclusions. Of the clinical research that has been done, almost all of it is in the form of pilot studies (e.g. [15-17]) that focus on the potential of a particular technology prototype rather than trying to prove its real-world efficacy. One study on a prototype system similar to YouGrabber shows improvements in speed of recovery in acute stroke patients [18]. Other research has been done on direct use of video game systems, e.g. the Nintendo Wii and Sony EyeToy [19-22], which focus methods for exploiting existing technologies. These studies generally show potential benefits of using conventional video games, but with disadvantages including lack of targeting of movement training to the needs of patients and inappropriate game difficulty. The pilot clinical studies clearly indicate the potential of YouGrabber to have a positive impact on training intensity and thus patient recovery of motor function.

YouGrabber, a novel virtual reality-based upper limb rehabilitation system, partially automates therapy to increase therapist efficiency at moderate cost. It also trains patients three times as intensively as conventional therapy, potentially leading to improved patient outcomes. YouGrabber is the only product on the market which can simultaneously train both arm and hand/finger function. To date, most of the clinical evidence for YouGrabber has been for paediatric patients, plus single-case studies documenting the efficacy of the system on stroke patients and there is a lack of strong clinical evidence of the therapeutic efficacy [23]. YouGrabber, for example, has been tested and evaluated in two chronic stroke patients with more than three years after stroke. Although YouGrabber showed promising effects regarding increased affected limb use and function in activities of daily living (ADL) [24] and also in paediatric cerebral palsy patients, the number of patients evaluated is too small to provide strong experimental support.

### 2.1 Research question, hypothesis, and aim

The study focuses on the evaluation of the YouGrabber efficacy compared to conventional therapy in an outpatient setting.

**Research question:** Do patients after stroke in the YouGrabber training group show higher post-intervention performance in the Box and Block Test (BBT) compared to patients in the conventional therapy group?

**Hypothesis:** $H_0$: We hypothesis that there will be no group differences after 16 training sessions or after the three month follow-up period.
$H_1$: We hypothesis that there will be a group difference after the 16 training sessions and after the three month follow-up period.

**Aim:** The aim of the project is to design and implement a single-blinded, randomised controlled multi-centre trial comparing YouGrabber training and conventional therapy in patients after stroke.

## 3 Methods

### 3.1 Study design and measurement events

The patient study is a phase III trial designed as a single-blinded, randomised, controlled multi-centre trial with repeated measurement events (ME). Patients will be evaluated by a blinded assessor on five occasions: twice within two weeks at baseline before intervention start (BL, T0), once after eight treatment sessions (T1), once after the intervention (T2), and once after a two month follow-up period (FU). Figure 1 illustrates the study overview.

### 3.2 Criteria for halting the trial

Until today, the YouGrabber system has been used for more than two years with over 100 patients in different acute hospitals and rehabilitation clinics. So far, no adverse events were reported. However, the study will be halted if one of the following criteria will be fulfilled:

- More than three patients in the experimental group report a sudden onset or an increase of shoulder pain during or just after therapy that is highly likely to be attributable to the use of YouGrabber, and which does not immediately cease after stopping therapy (evaluated with level ≤3 of the Chedoke-McMaster Stroke Assessment subscale pain)
- More than 50% of patients (min. 5 patients) in the experimental group report severe cyber-sickness during YouGrabber training, which persists after training is halted.
- Patients in the experimental group show an unexpected decrease in motor function (change of three or more levels in the Chedoke-McMaster Stroke Assessment) indicating a highly significant difference between therapies with YouGrabber compared to conventional therapy.
- Epileptic seizures in at least two patients induced directly while using YouGrabber.

Patients reporting the criteria mentioned above will be evaluated by the physician on duty and will be assessed and followed up for the originally planned study duration.

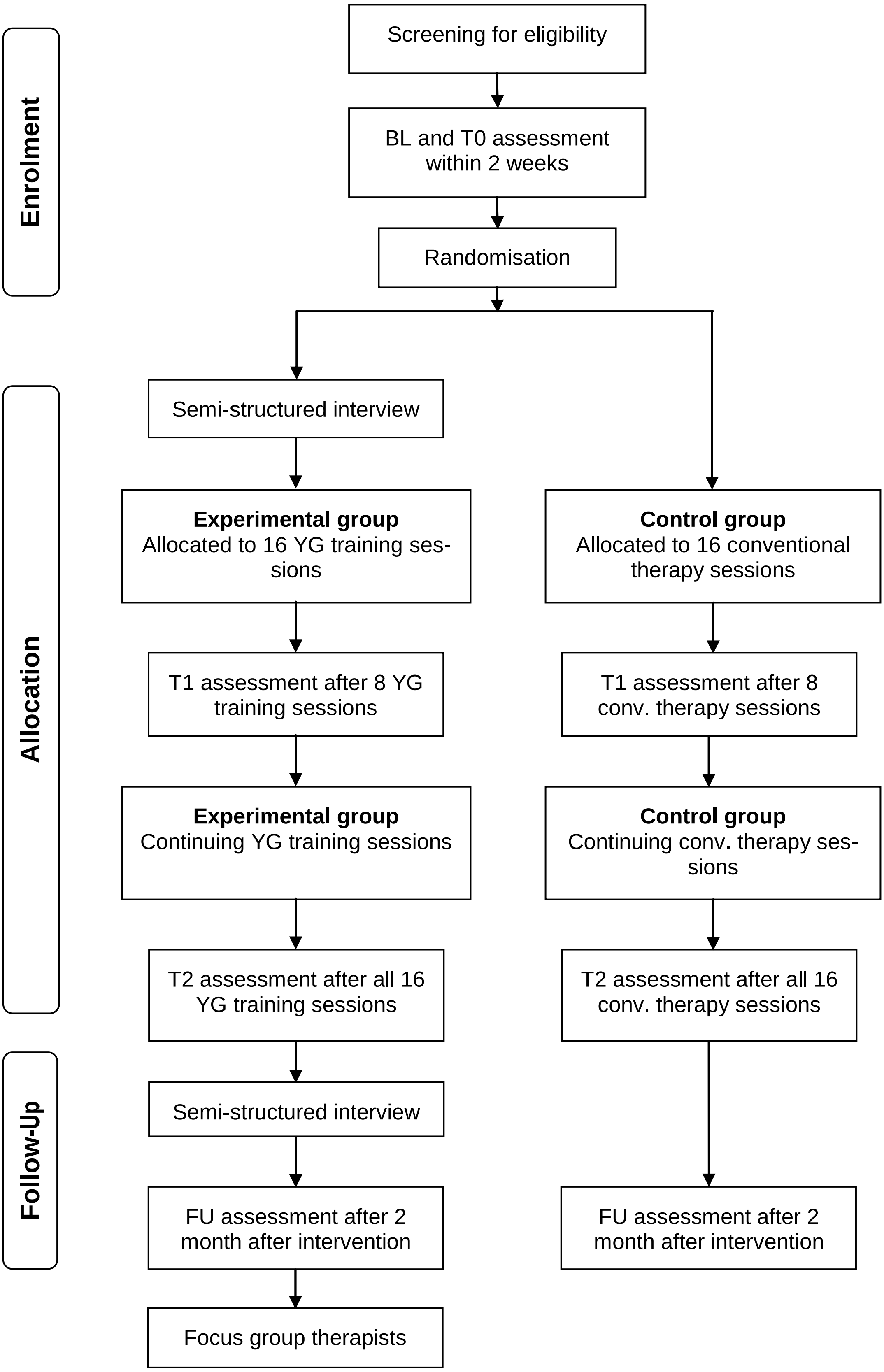


Legend: BL=Baseline, T0=pre-intervention, T1=Measurement after 8 treatment sessions, T2=Measurement event after intervention, FU=Measurement event after 2-month follow-up period, YG=YouGrabber.

Figure 1: Study overview.

## 3.3 Patient selection criteria and recruitment

Patients will be eligible for study participation if they fulfil the following selection criteria in Table 1:

Table 1: Patient selection criteria

| Inclusion criteria | Exclusion criteria |
|---|---|
| • ≥ 6 months after first-ever stroke (ischemic, haemorrhagic).<br>• Able to sit in a normal chair without armrests and without support of the back rest.<br>• Persistent motor deficit of the arm and hand confirmed by the Chedoke-McMaster Stroke Assessment (CMSA) subscale arm level x≥3 and subscale hand level x≥2. The difference between both subscales has to be 2 levels or more.<br>• Able to score at least one in the Box and Block Test (main outcome measure). | • Previous or current other functional deficits of arm and hand motor function not due to stroke.<br>• Severe cognitive deficits MMSE ≤ 20.<br>• Severe spatial-visual disorders, e.g. severe visual neglect confirmed by a Line-Bisection-Test.<br>• History of epileptic seizures triggered by visual stimuli (e.g. television, video games) within the last six months. |

The study's patient recruitment strategy includes different approaches:

1. Patients will be recruited from the **clinics' inpatient or outpatient department** by physicians, therapists, and nurses.
2. Patients will be recruited from the **clinic's patient database**. Data sets will be screened for study selection criteria by the responsible clinic personnel. If patients will be eligible they will receive an information letter describing the study and including a response sheet and envelope. If they are interested in participating, patients can contact the study personnel in the responsible clinic by phone, postal mail, or email.
3. Patients will be recruited by a study information flyer provided on each **clinic's homepage** and by **patient self-help groups.** If patients are interested in participating they can contact the study personnel in the responsible clinic by phone, postal mail, or email.

## 3.4 Randomisation and group allocation

Patients will be randomly allocated to either the experimental group (EG) or the control group (CG) after the second ME (T0). Group allocation will be based on a computer-generated randomisation list (one for each centre, (MATLAB, 2007b, Mathworks Inc., USA) created by a researcher not involved into the study. Randomisation lists and corresponding token will be stored in the clinics' pharmacy. Patients will draw a token before the first therapy session. The token will be marked and stored until study finalisation in the pharmacy.
Group allocation will remain concealed for the independent assessor until study finalisation. Patients and treating therapists will be reminded not to talk about patient's group allocation with other therapists or participants.

## 3.5 Study interventions

Patients in both study groups (EG, CG) will receive the same amount of 16 sessions lasting for 45 minutes each. During each therapy appointment patients can decide to stop the training at any time.
Patients allocated to EG will have the opportunity to participate in two semi-structured interviews to evaluate their expectations and experiences with the virtual reality therapy with YouGrabber (Figure 1). Interview participation will be voluntary. Aim, methods, and data analysis of the semi-structured interview will be described below.

### 3.5.1 Experimental group (EG):

Patients in EG will sit in front of the YouGrabber-System (Figure 2). They will wear hand gloves with attached sensors to measure finger movements of the thumb, index finger, middle finger, wrist (bending, extending), and lower upper limb (pronation, supination). Movements will be displayed on the monitor in real-time.
YouGrabber offers a variety of training applications for different movements and at different intensity levels. The settings for each session can be tailored to the patients' preferences or movement aim. Together with the therapist, patients can also select one of three modes to control the on-screen finger and arm movements: a) using the real arm/hand movements, b) mirroring of the real movements of one arm/hand, and c) following the movements of one arm/hand. A detailed description can be found in the YouGrabber system manual in the Appendix.
After the second YouGrabber training session patients should have tested all training applications and all three modes of finger/hand movements. In the remaining 14 sessions therapists are asked to select at least three training applications for each training session and two different movement modes with adapted settings to the patients' needs.

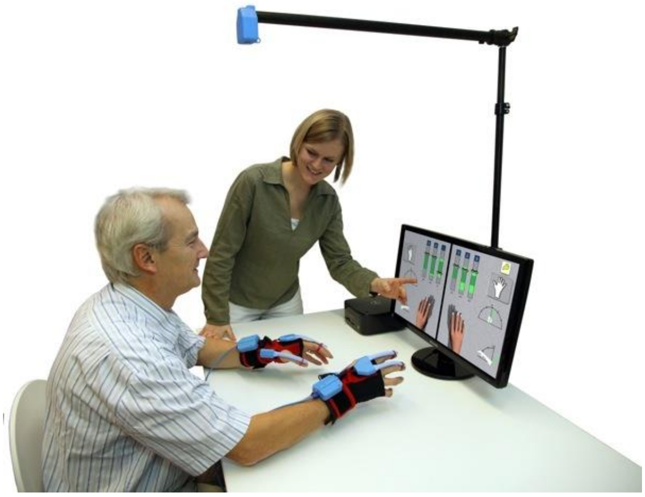

Figure 2: YouGrabber training set up.

Control group (CG):

Patients in CG will receive conventional physiotherapy or occupational therapy. The therapy content will focus on a task-related upper limb treatment in a sitting or lying position. Therapy will be tailored for each patient depending on the motor function level.

Therapy in an upright position should be avoided as well as gait or stair climbing training. All manual techniques, therapy material, and objects of ADL are allowed for treatment. Additional electrical or mechanical therapy devices (e.g. help arm systems, splints) should be avoided.

### 3.5.2 Semi-structured interviews

**Aim:** The aim of the interviews is the exploration and description of the patients' and treating therapists' experiences and expectations during the intervention with the YouGrabber training system using virtual reality. It is of high relevance to consider the patient and therapist perspective, in particular if new therapy systems will be evaluated regarding their effectiveness. The gained knowledge can be used to improve the training system and to formulate treatment guidelines.

**Methodological procedure:** Interviews will be conducted with patients in EG who worked with the YouGrabber system: patients - semi-structured one-to-one interviews before and after the intervention, therapists - focus group, after the intervention. Interviews will be recorded with two digital voice recorders. Interview content will be transcribed verbatim.
**Data analysis:** Data analysis is based on a descriptive phenomenological approach without data or opinion interpretation [25]. Ten to 15 patients will be sufficient to explore the phenomenon under investigation [26]. Data analysis will include several steps:
1. Step - TRANSCRIPTION: Interview content will be verbatim described.
2. Step - CONDENSATION: Patients' and therapists' quotes will be summarised to highlight the main statement.
3. Step – CODING and CATEGORISATION: Based on the thematic analysis categories and codes will be created to sort patients' and therapists' statements [27]. This can be done with the help of a qualitative data analysis tool, e.g. NVivo or Atlas.ti.

## 3.6 Outcome parameters and related outcome measures

Table 2 provides an overview on all outcome measures, time needed to perform the measurement, and measurement events. Each assessment will be described in detail below.

Table 2: Overview outcome measures.

| Assessment | Abbreviation | Category | Time needed in min | Total amount | Measurement events | | | | |
|---|---|---|---|---|---|---|---|---|---|
| | | | | | BL | T0 | T1 | T2 | FU |
| Box and Block Test* | BBT | Performance measure | 15 | 5x | X | X | X | X | X |
| Chedoke-McMaster Stroke Assessment | CMSA | Motor impairment | 20 | 5x | X | X | X | X | X |
| Chedoke McMaster Arm and Hand Activity Inventory | CAHAI | Activity (ADL) | 45 | 5x | X | X | X | X | X |
| Extended Barthel Index | EBI | Independence | 10 | 1x | X | | | | |
| Edinburgh Handedness Inventory | EHI | N/A | 5 | 1x | X | | | | |
| Mini Mental State Examination | MMSE | Cognitive assessment | 15 | 1x | X | | | | |
| Line Bisection Test | LBT | Neglect assessment | 10 | 5x | X | X | X | X | X |
| Active range of motion of affected fingers | ROM | YouGrabber | 15 | 5x | X | X | X | X | X |
| Stroke Impact Scale | SIS | Impact of stroke on ADL, mobility, emotion, memory, strength, communication | 30 | 5x | X | X | X | X | X |

**Legend:** *=primary outcome measure, min=minutes, BL=Baseline, T0=pre-intervention, T1=after 8 treatment sessions, T2=after 16 treatment session, FU=Follow-up after 2 months after study treatment finalisation, N/A=not applicable.

### 3.6.1 Primary outcome: hand dexterity

Change of hand dexterity between T0 and T2 is the primary outcome of interest. It will be measured with the **Box and Block Test** that is described by Mathiowetz et al. in 1985 [28]. The test is easy to administer and fast to perform. It has been used in patients after stroke, with Multiple Sclerosis, and Traumatic Brain Injury [29]. Patients will be asked to grasp small wooden cubes and move them from one side of the box to the other side as fast as possible within 60 seconds. The BBT is a reliable and valid assessment, and provides norm data for healthy individuals for age groups starting with 20 to older than 75.

### 3.6.2 Secondary outcomes:

Upper limb ADL, motor and cognitive function are secondary outcomes. They will be assessed objectively with the Chedoke-McMaster Stroke Assessment (CMSA), the Chedoke Arm and Hand Activity Inventory (CAHAI), Line Bisection Test (LBT) and active range of motion (ROM), and subjectively with the Stroke Impact Scale (SIS).

The **CMSA** has been developed by Gowland et al. in 1995 and evaluates physical impairment and activity level of stroke patients [30]. In this study the impairment subscales for hand and arm function will be used. Scales will be scored on a seven-point scale (1 = needs total assistance, 7 = independent) according to seven stages of motor recovery [31]. Additionally, shoulder pain of the affected body side will be evaluated on the same seven-point scale using a further subscale. The CMSA was shown to be a valid and reliable assessment [30, 32]. The higher the scoring the more independent is the patient.

The **CAHAI** has been developed by Barreca et al. in 2004 [33-35]. The CAHAI contains 13 real-life items scored from one to seven (highest score), e.g. put toothpaste on toothbrush. Scoring represents the independence of patients to perform stabilisation or manipulation in ADL with the affected upper limb. Score one represents total dependence from another person, whereas score seven shows patient's independence without time or safety concerns, necessary splints or devices. Quality of reliability and validity has been evaluated [35-36]. The higher the scoring the more independent is the patient.

The **LBT** is a paper and pencil test to evaluate presence of an unilateral spatial neglect (USN) [37]. Patients are asked to mark the centre of a drawn line on paper with the pencil. This is repeated for 18 lines on a sheet of paper. More than 60 millimetre deviation from the centre indicate the presence of USN.

Active **ROM** will be assessed with YouGrabber. Similar to the YouGrabber training session patients will sit in front of the monitor and they will wear both gloves that are connected to the computer (Figure 2). Single finger and wrist bending and extension, and pronation and supination of the lower arm will be measured.

The **SIS** is a questionnaire comprising questions regarding the impact of stroke on physical function, emotion, memory, communication, and social participation. The SIS has been developed by Duncan and colleagues in 2001 and modified over the recent years [38]. The current version 3.0 consists of 59 questions that should be administered in a one-to-one interview. Patients can rate the level of the stroke impact on a five-point Likert scale. Reliability, validity, and sensitivity to change have been evaluated for the version 2.0 [39]. Official translations have been produced for 14 languages, e.g. German and French. The higher the scoring the less affected feels the patient.

### 3.6.3 Further assessments and evaluations

The Mini-Mental State Examination [40], the Edinburgh Handedness Inventory [41], and the Extended Barthel Index [42] will be used for patient evaluation and descriptive purposes at BL only.

## 3.7 Sample size and statistical analyses

Based on the study on the efficacy of YouGrabber for children with cerebral palsy in the rehabilitation clinic of Affoltern-am-Albis [43] a power analysis and sample size calculation for the current study were performed with the G-Power software 3.0 [44]. The BBT (primary outcome measure) showed an effect size (rho) of 0.98. Assuming a similar effect size for adult stroke patients, to

achieve a significance level (p-value) of 0.05 per group 26 patients would be needed. Considering a drop out rate of 15% a total of 60 patients across participating centres will be recruited.

Data will be analysed qualitatively and quantitatively for each patient separately. Changes will be given as total number or percentage. Related to the null hypothesis group comparisons between EG and CG will be analysed using ANOVA after data have been tested for normal distribution. It is intended to perform an intention-to-treat analyses. If necessary, an additional per protocol analysis will be carried out.
Patients' training settings (nominal and interval data) will be analysed with frequencies and percentages to describe therapy content and provide recommendations for further therapy settings.

All study personnel will try to avoid patient drop outs. However, patients can leave the study at any time without reason. Conducted data of those patients will be included in the final analysis. Missing data will be replaced using two different approaches: a) with the last available value carried forward method, and b) adding or subtracting the mean difference value of other patients of the respective group. Analysis with both approaches will be performed.

## 3.8 Ethical considerations and reporting of adverse events

The YouGrabber system is a commercial training system using virtual reality to improve upper limb motor function. It has been available in a beta version for test clinics since 2010 and has been used in rehabilitation institutions and pilot clinical studies with no safety-related incidents. Nevertheless, it might occur that patients' experience sadness, frustration, or have the impression that they cannot perform the training tasks due to their reduced motor function. It is expected that study participation will take a lot of patients' time. In exchange for the patients' time exposure to the current study:

- Patients will be assessed on a regular basis to evaluate their rehabilitation process.
- Patients will have an intensive therapy schedule with four therapy sessions per week for four weeks.
- Patients will have the opportunity to get to know the innovative therapy system YouGrabber that uses virtual reality.
- Study participation is free. Patients will receive CHF 10 per study visit to reimburse their travel costs.

It is expected that patients will receive accompanying therapies, e.g. occupational therapy, speech and language therapy. If possible, accompanying therapies related to upper limb motor function shall be postponed or take place on a reduced basis for the four week intervention period only.

The study will be carried out in accordance with the protocol, guidelines on Good Clinical Practice and current national and international valid legal provisions. Any adverse event will be reported to the responsible ethics committee directly and will be mentioned in intermediate and final study reports.

The study will be registered with the trials database clinicaltrials.gov.

## 3.9 Study insurance and data safety

For the current study the University of Zurich has arranged an insurance (Nationale Suisse, + contract number XXX) for the participating study centres covering all study related damage.

All study related patient data will be entered directly into the anonymised case report forms or assessment scoring sheets. Study related original patient documents will remain in the responsible clinic and will be archived for 10 years. After that period patient documents will be destroyed in accordance with the clinic's data destruction guidelines. Only anonymised electronic data or hard copies will be send to the University of Zurich for data analyses.

Training data collected by the YouGrabber system will be saved in anonymised form under the patient's study ID, with no other identifying information. Data backup and synchronization will occur using SugarSunc. The data transfer protocol used in Sugarsync works with secure socket layer technology (SSL) and is certified to conform to the U.S. - Swiss Safe Harbor framework (https://www.sugarsync.com/privacy.html). This secure data transfer and storage framework is also approved by Swissmedic for use in clinical studies.

For external audits, monitoring events, and inspections authorised personnel (ethics committee, CTI representatives) will have direct access to all study data in each clinic. Patient data will be treated as strictly confidential and all documents will remain in the responsible clinic.

### 3.10 Quality control and quality assurance: description of measures

To achieve and remain a high quality standard the following measures will apply:

- Comprehensive training for all study therapists to use the YouGrabber therapy system before study start.
- Comprehensive training for all study therapists to perform all necessary outcome measures before study start.
- Three refresher training days for YouGrabber training and assessment performance during the study duration
- Study management updates for all centres on a regularly basis (monthly or bimonthly).
- Daily telephone and email reachabiliy for technical support.

Furthermore, up to three monitoring events will be performed for each participating study centre. An authorised person from the sponsor, who is not involved in patient treatment or assessments will visit the clinics and inspect data handling and patient organisation. Up to five randomly selected patient data files will be inspected.

All study patients will also have the opportunity to receive a summary of the study results.

## 4 References


1. Truelsen T, Piechowski-Jozwiak B, Bonita R, Mathers C, Bogousslavsky J, Boysen G: **Stroke incidence and prevalence in Europe: a review of available data**. *Eur J Neurol* 2006, **13**(6):581-598.
2. **Stroke Statistics** [http://www.strokecenter.org/patients/about-stroke/stroke-statistics/]
3. **Gesundheitszustand und Krankheiten - Daten, IndikatorenÜbersicht chronische Krankheiten** [http://www.bfs.admin.ch/bfs/portal/de/index/themen/14/02/01/key/02.htm]
4. Gaggioli A, Meneghini A, Morganti F, Alcaniz M, Riva G: **A strategy for computer-assisted mental practice in stroke rehabilitation**. *Neurorehab Neural Re* 2006, **20**(4):503-507.
5. Holden MK: **Virtual environments for motor rehabilitation: review**. *Cyberpsychol Behav* 2005, **8**(3):187-211; discussion 212-189.
6. Buccino G, Binkofski F, Riggio L: **The mirror neuron system and action recognition**. *Brain Lang* 2004, **89**(2):370-376.
7. Buccino G, Binkofski F, Fink GR, Fadiga L, Fogassi L, Gallese V, Seitz RJ, Zilles K, Rizzolatti G, Freund HJ: **Action observation activates premotor and parietal areas in a somatotopic manner: an fMRI study**. *Eur J Neurosci* 2001, **13**(2):400-404.
8. Fadiga L, Fogassi L, Pavesi G, Rizzolatti G: **Motor facilitation during action observation: a magnetic stimulation study**. *Journal of neurophysiology* 1995, **73**(6):2608-2611.

9. Braun SM, Beurskens AJ, Borm PJ, Schack T, Wade DT: **The effects of mental practice in stroke rehabilitation: a systematic review**. *Arch Phys Med Rehabil* 2006, **87**(6):842-852.
10. Sharma N, Pomeroy VM, Baron JC: **Motor imagery - A backdoor to the motor system after stroke?** *Stroke* 2006, **37**(7):1941-1952.
11. Zimmermann-Schlatter A, Schuster C, Puhan M, Siekierka E, Steurer J: **Efficacy of motor imagery in post-stroke rehabilitation: a systematic review**. *J Neuroeng Rehabil* 2008, **5**(8).
12. Fadiga L, Buccino G, Craighero L, Fogassi L, Gallese V, Pavesi G: **Corticospinal excitability is specifically modulated by motor imagery: a magnetic stimulation study**. *Neuropsychologia* 1999, **37**(2):147-158.
13. Stefan K, Cohen LG, Duque J, Mazzocchio R, Celnik P, Sawaki L, Ungerleider L, Classen J: **Formation of a motor memory by action observation**. *J Neurosci* 2005, **25**(41):9339-9346.
14. Laver KE, George S, Thomas S, Deutsch JE, Crotty M: **Virtual reality for stroke rehabilitation**. *Cochrane Database Syst Rev* 2011(9):CD008349.
15. Adamovich S, Merians A, Boian R, Tremaine M, Burdea G, Recce M, Poizner H: **A virtual reality (VR)-based exercise system for hand rehabilitation post stroke**. *Presence* 2005, **14**:161 - 174.
16. King M, Hale L, Pekkari A, Persson M, Gregorsson M, Nilsson M: **An affordable, computerised, table-based exercise system for stroke survivors**. *Disabil Rehabil Assist Technol* 2010, **5**(4):288-293.
17. Sampson M, Shau YW, King MJ: **Bilateral upper limb trainer with virtual reality for post-stroke rehabilitation: case series report**. *Disabil Rehabil Assist Technol* 2012, **7**(1):55-62.
18. da Silva Cameirao M, Bermudez IBS, Duarte E, Verschure PF: **Virtual reality based rehabilitation speeds up functional recovery of the upper extremities after stroke: a randomized controlled pilot study in the acute phase of stroke using the rehabilitation gaming system**. *Restor Neurol Neurosci* 2011, **29**(5):287-298.
19. Huber M, Rabin B, Docan C, Burdea GC, AbdelBaky M, Golomb MR: **Feasibility of modified remotely monitored in-home gaming technology for improving hand function in adolescents with cerebral palsy**. *IEEE Trans Inf Technol Biomed* 2010, **14**(2):526-534.
20. Mouawad MR, Doust CG, Max MD, McNulty PA: **Wii-based movement therapy to promote improved upper extremity function post-stroke: a pilot study**. *J Rehabil Med* 2011, **43**(6):527-533.
21. Saposnik G, Teasell R, Mamdani M, Hall J, McIlroy W, Cheung D, Thorpe KE, Cohen LG, Bayley M: **Effectiveness of virtual reality using Wii gaming technology in stroke rehabilitation: a pilot randomized clinical trial and proof of principle**. *Stroke* 2010, **41**(7):1477-1484.
22. Yavuzer G, Senel A, Atay MB, Stam HJ: **"Playstation eyetoy games" improve upper extremity-related motor functioning in subacute stroke: a randomized controlled clinical trial**. *Eur J Phys Rehabil Med* 2008, **44**(3):237-244.
23. Ramachandran VS, Hirstein W: **The perception of phantom limbs. The D. O. Hebb lecture**. *Brain* 1998, **121 ( Pt 9)**:1603-1630.
24. Heinrichs A, Schuster C, Eng K, Keisker B, Holper L, Chevrier E, Pyk P, Kiper D: **Interactive bimanual virtual mirror therapy on chronic stroke: two single case studies**. In *WCPT 2011; Amsterdam*; 2011.
25. Speziale HJ, Carpenter DR: *Qualitative research in nursing: Advancing the humanistic imperative*. 4th edition. Philadelphia: Lippincott Williams & Wilkins; 2007.
26. Starks H, Trinidad SB: **Choose your method: a comparison of phenomenology, discourse analysis, and grounded theory**. *Qual Health Res* 2007, **17**(10):1372-1380.
27. Braun V, Clarke V: **Using thematik analysis in psychology**. *Qualitative research in psychology* 2006, **3**:77-101.
28. Mathiowetz V, Volland G, Kashman N, Weber K: **Adult norms for the Box and Block Test of manual dexterity**. *Am J Occup Ther* 1985, **39**(6):386-391.

29. Connell LA, Tyson SF: **Clinical reality of measuring upper-limb ability in neurologic conditions: a systematic review**. *Arch Phys Med Rehabil* 2012, **93**(2):221-228.
30. Gowland C, VanHullenaar S, Torresin W, Moreland J, Vanspall B, Barreca S, Ward M, Huijbregts M, Stratford P, Barclay-Goddard R: *Chedoke-McMaster Stroke Assessment: development, validation, and administration manual*. Hamilton (ON): School of Rehabilitation Science, McMaster University; 1995.
31. Gowland CA: **Staging motor impairment after stroke**. *Stroke* 1990, **21**(9 Suppl):II19-21.
32. Gowland C, Stratford P, Ward M, Moreland J, Torresin W, Van Hullenaar S, Sanford J, Barreca S, Vanspall B, Plews N: **Measuring physical impairment and disability with the Chedoke-McMaster Stroke Assessment**. *Stroke* 1993, **24**(1):58-63.
33. Barreca S, Gowland CK, Stratford P, Huijbregts M, Griffiths J, Torresin W, Dunkley M, Miller P, Masters L: **Development of the Chedoke Arm and Hand Activity Inventory: theoretical constructs, item generation, and selection**. *Top Stroke Rehabil* 2004, **11**(4):31-42.
34. Barreca S, Stratford P, Masters L, Lambert CL, Griffiths J, McBay C: **Validation of Three Shortened Versions of the Chedoke Arm and Hand Activity Inventory**. *Physiother Can* 2006, **58**:148-156.
35. Barreca SR, Stratford PW, Lambert CL, Masters LM, Streiner DL: **Test-retest reliability, validity, and sensitivity of the Chedoke arm and hand activity inventory: a new measure of upper-limb function for survivors of stroke**. *Arch Phys Med Rehabil* 2005, **86**(8):1616-1622.
36. Schuster C, Hahn S, Ettlin T: **Objectively-assessed outcome measures: a translation and cross-cultural adaptation procedure applied to the Chedoke McMaster Arm and Hand Activity Inventory (CAHAI)**. *BMC Med Res Methodol* 2010, **10**:106.
37. Plummer P, Morris ME, Dunai J: **Assessment of unilateral neglect**. *Phys Ther* 2003, **83**(8):732-740.
38. Duncan PW, Wallace D, Studenski S, Lai SM, Johnson D: **Conceptualization of a new stroke-specific outcome measure: the stroke impact scale**. *Top Stroke Rehabil* 2001, **8**(2):19-33.
39. Duncan PW, Wallace D, Lai SM, Johnson D, Embretson S, Laster LJ: **The stroke impact scale version 2.0. Evaluation of reliability, validity, and sensitivity to change**. *Stroke* 1999, **30**(10):2131-2140.
40. Folstein MF, Folstein SE, McHugh PR: **"Mini-mental state". A practical method for grading the cognitive state of patients for the clinician**. *Journal of psychiatric research* 1975, **12**(3):189-198.
41. Oldfield RC: **The assessment and analysis of handedness: the Edinburgh inventory**. *Neuropsychol* 1971, **9**(1):97-113.
42. Jansa J, Pogacnik T, Gompertz P: **An evaluation of the Extended Barthel Index with acute ischemic stroke patients**. *Neurorehabil Neural Repair* 2004, **18**(1):37-41.
43. van Hedel HJA, Wick K, Eng K, Meyer-Heim A: **Improving dexterity in children with cerebral palsy: Preliminary results of a randomised trial evaluating a glove based VR-system**. Affoltern am Albis: Children University Hospital; 2011.
44. Faul F, Erdfelder E, Lang A-G, Buchner A: **GPower**. 3.0.8 edition. Düsseldorf: Heinrich Heine Universität; 2007.
