# Supplementary material for: Effect of a four-week virtual reality-based training versus conventional therapy on upper limb motor function after stroke: A multicenter parallel group randomized trial
Source: PLoS One. 2018 Oct 24;13(10):e0204455. doi: 10.1371/journal.pone.0204455 (PMC6200191; doi:10.1371/journal.pone.0204455)
Supplement: S1 Table — Effect sizes range from 0.13 to 0.52 for upper limb related subscales, e.g. strength, ADL, and hand function, excluding subscale mobility. (DOCX) [file pone.0204455.s003.docx]

**Supplementary table S2:** Effect sizes.

|  | YouGrabber Training n=22 | | | | Conventional therapy n=32 | | | |  |
| --- | --- | --- | --- | --- | --- | --- | --- | --- | --- |
|  | Pre-test | Post test | SD pre-test12 | Effect size | Pre-test | Post test | SD pre-test | Effect size | |
| BBT affected side | 23.3 | 25.6 | 15.6 | 0.15 | 19.7 | 22.3 | 16.5 | 0.16 | |
| CAHAI | 67.7 | 73.3 | 18.8 | 0.30 | 64.2 | 67.1 | 23.2 | 0.13 | |
| SIS strength | 58.5 | 66.8 | 16.1 | 0.52 | 57.4 | 60.2 | 13.6 | 0.21 | |
| SIS ADL | 77.4 | 84.0 | 18.1 | 0.36 | 74.9 | 78.7 | 12.1 | 0.31 | |
| SIS mobility | 86.6 | 88.1 | 14.4 | 0.10 | 83.3 | 84.5 | 14.2 | 0.08 | |
| SIS hand function | 59.1 | 68.0 | 25.5 | 0.35 | 46.3 | 59.1 | 29.0 | 0.44 | |
| SIS stroke recovery | 56.5 | 64.2 | 17.7 | 0.44 | 59.8 | 62.2 | 16.0 | 0.15 | |
| SIS mobility index | 4.1 | 4.4 | 0.7 | 0.43 | 3.9 | 4.1 | 0.6 | 0.33 | |

Legend: Pre- and post-test values represent means. Effect sizes range 0.13 bis 0.52 for upper limb related subscales, e.g. strength, ADL, and hand function, excluding subscale mobility.
