# Supplementary figures and images for: Effect of a four-week virtual reality-based training versus conventional therapy on upper limb motor function after stroke: A multicenter parallel group randomized trial
Source: PLoS One. 2018 Oct 24;13(10):e0204455. doi: 10.1371/journal.pone.0204455 (PMC6200191; doi:10.1371/journal.pone.0204455)

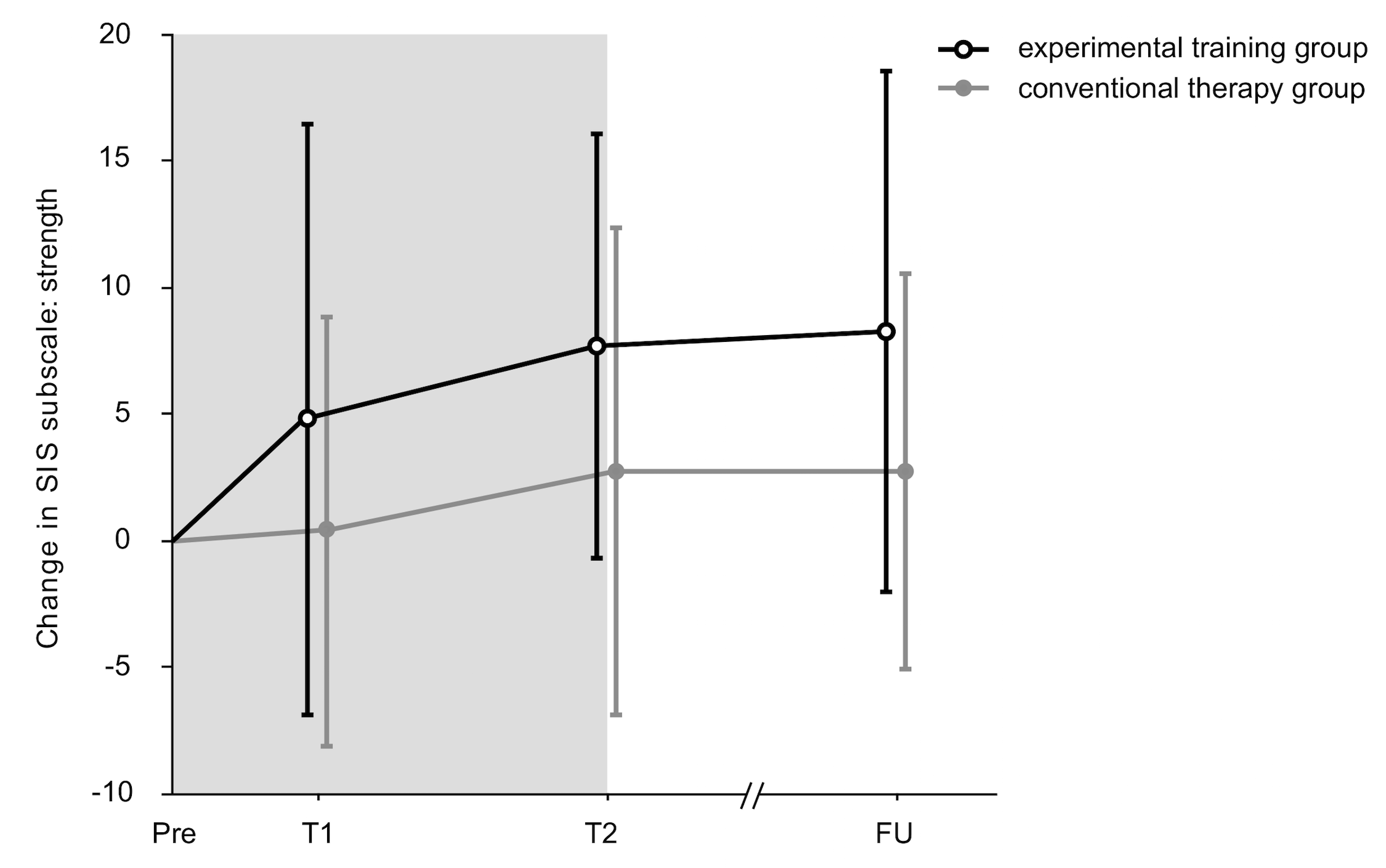

Supplement: S1 Fig — Pre = Pre-intervention, T1 = after 8 training sessions, T2 = after 16 training sessions, FU = follow-up after two months. (TIF) [file pone.0204455.s004.tif]

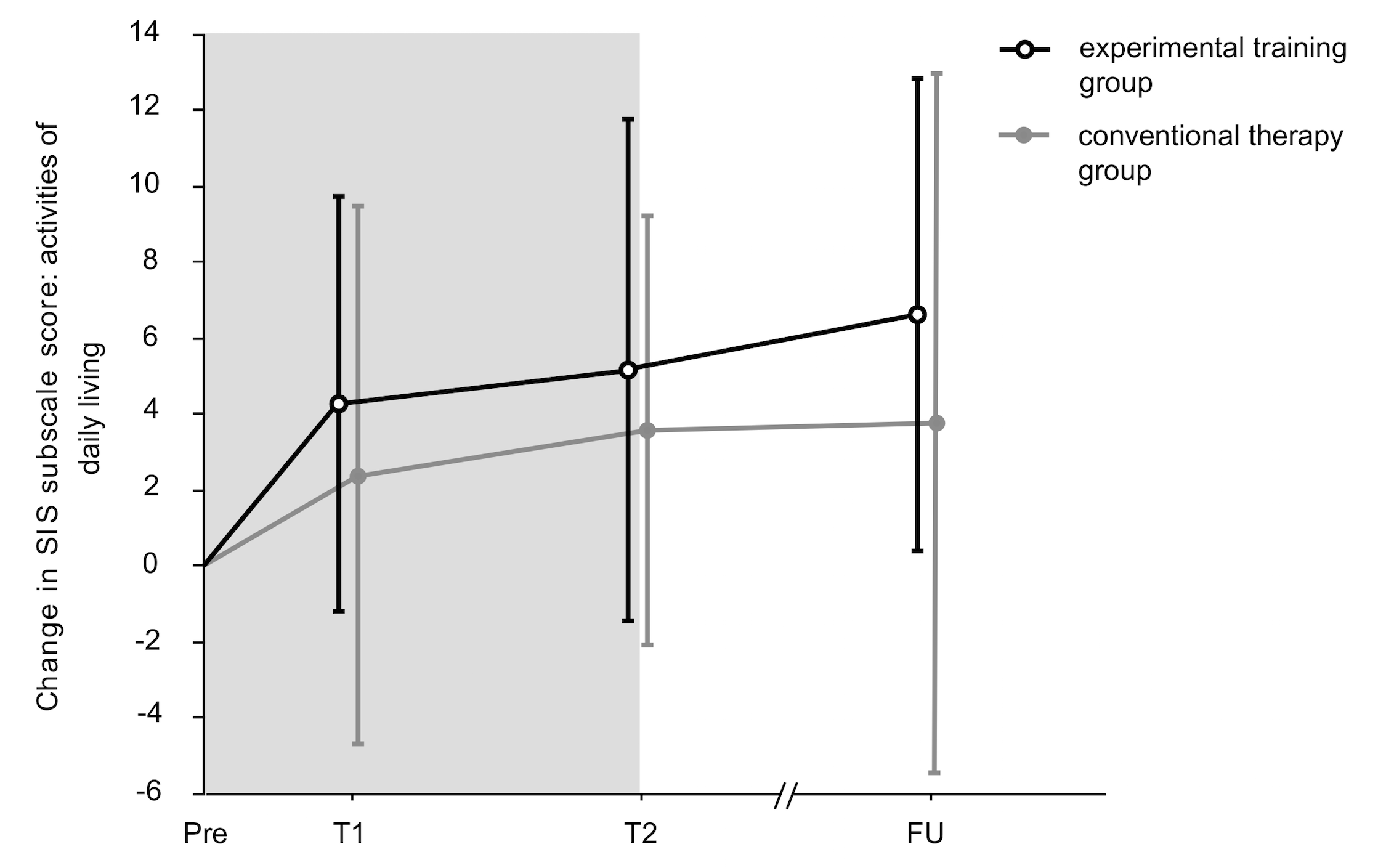

Supplement: S2 Fig — Pre = Pre-intervention, T1 = after 8 training sessions, T2 = after 16 training sessions, FU = follow-up after two months. (TIF) [file pone.0204455.s005.tif]

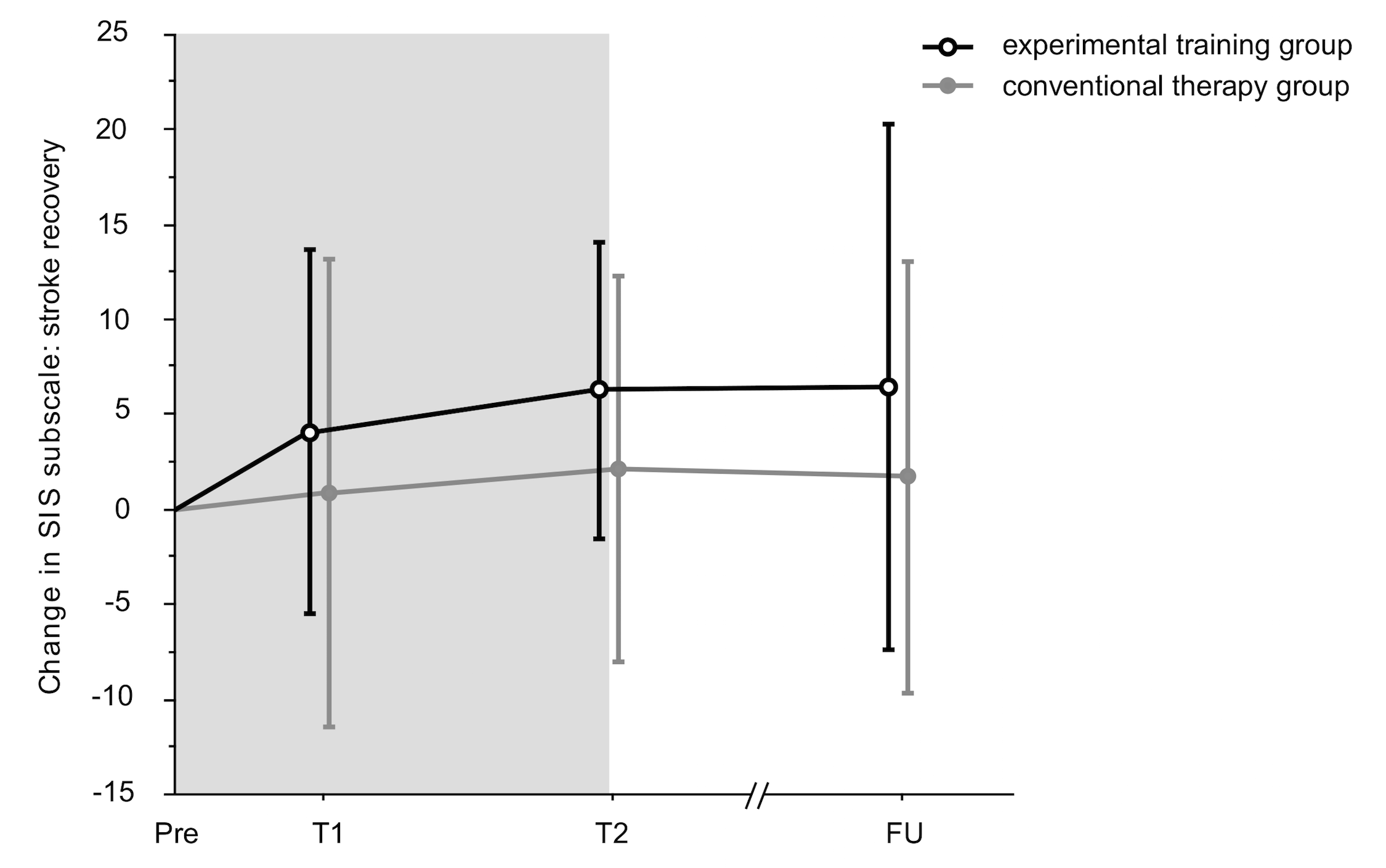

Supplement: S3 Fig — Pre = Pre-intervention, T1 = after 8 training sessions, T2 = after 16 training sessions, FU = follow-up after two months. (TIF) [file pone.0204455.s006.tif]

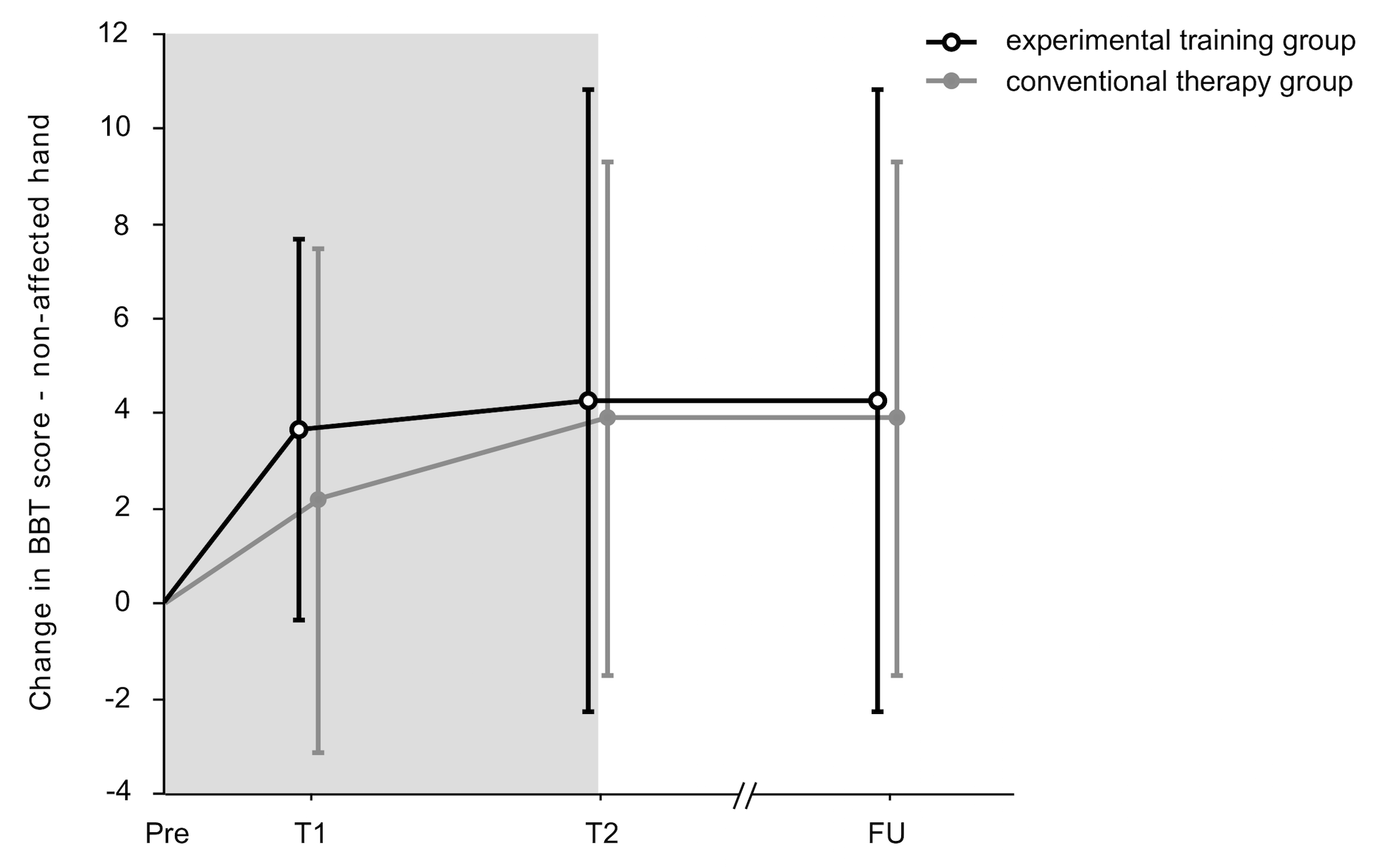

Supplement: S4 Fig — Pre = Pre-intervention, T1 = after 8 training sessions, T2 = after 16 training sessions, FU = follow-up after two months. (TIF) [file pone.0204455.s007.tif]
